# Supplementary material for: Social support, social network and salt-reduction behaviours in children: a substudy of the School-EduSalt trial
Source: BMJ Open. 2019 Jun 14;9(6):e028126. doi: 10.1136/bmjopen-2018-028126 (PMC6589018; doi:10.1136/bmjopen-2018-028126)
Supplement: Supplementary file 1 [file bmjopen-2018-028126supp001.pdf]

Online Supplementary Material

Figure S1. The association between the existence of friend ties and similarity in SRB score

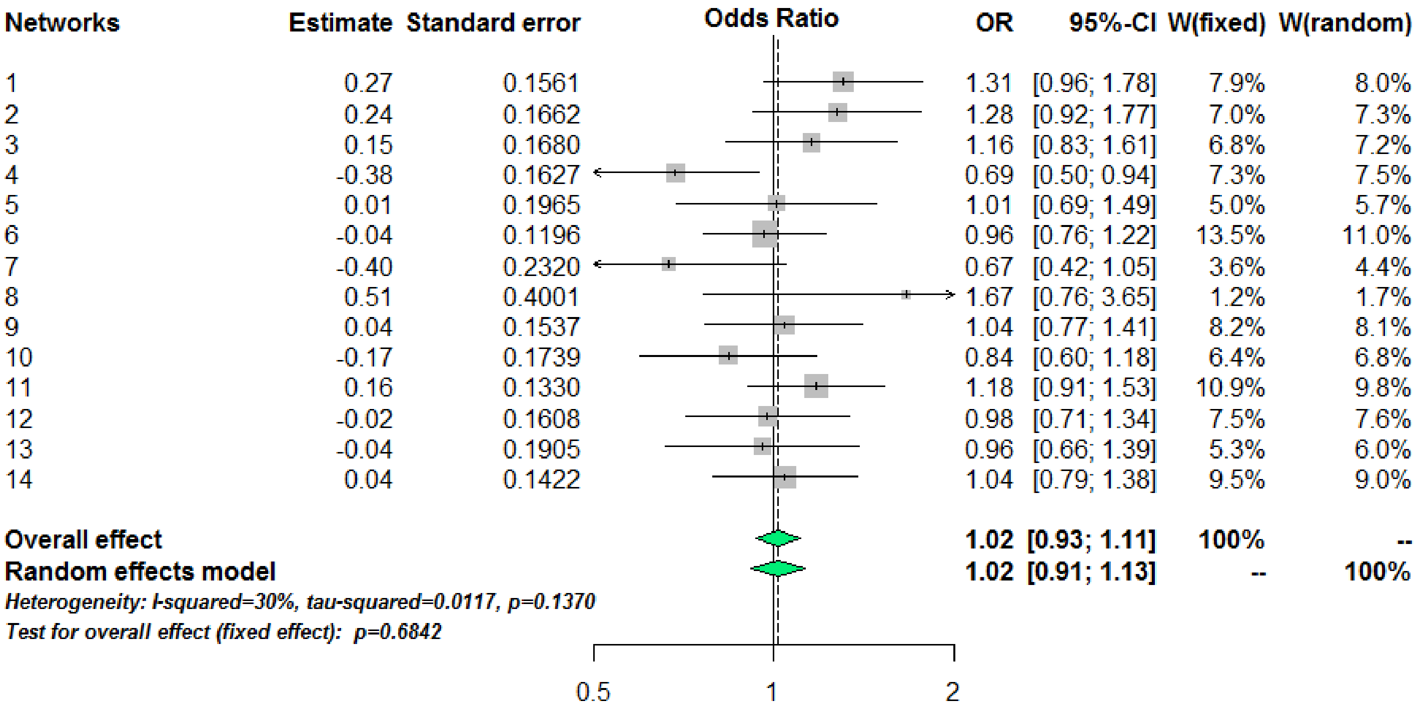

Networks	Estimate	Standard error	OR	95%-CI	W(fixed)	W(random)
1	0.27	0.1561	1.31	[0.96; 1.78]	7.9%	8.0%
2	0.24	0.1662	1.28	[0.92; 1.77]	7.0%	7.3%
3	0.15	0.1680	1.16	[0.83; 1.61]	6.8%	7.2%
4	-0.38	0.1627	0.69	[0.50; 0.94]	7.3%	7.5%
5	0.01	0.1965	1.01	[0.69; 1.49]	5.0%	5.7%
6	-0.04	0.1196	0.96	[0.76; 1.22]	13.5%	11.0%
7	-0.40	0.2320	0.67	[0.42; 1.05]	3.6%	4.4%
8	0.51	0.4001	1.67	[0.76; 3.65]	1.2%	1.7%
9	0.04	0.1537	1.04	[0.77; 1.41]	8.2%	8.1%
10	-0.17	0.1739	0.84	[0.60; 1.18]	6.4%	6.8%
11	0.16	0.1330	1.18	[0.91; 1.53]	10.9%	9.8%
12	-0.02	0.1608	0.98	[0.71; 1.34]	7.5%	7.6%
13	-0.04	0.1905	0.96	[0.66; 1.39]	5.3%	6.0%
14	0.04	0.1422	1.04	[0.79; 1.38]	9.5%	9.0%
**Overall effect**			**1.02**	**[0.93; 1.11]**	**100%**	**--**
**Random effects model**			**1.02**	**[0.91; 1.13]**	**--**	**100%**

Heterogeneity: I-squared=30%, tau-squared=0.0117, p=0.1370

Test for overall effect (fixed effect): p=0.6842

*Odds ratio of the existence of a friend tie associated with one-unit increase in the similarity of SRB score between each pair of children.

Supplementary Table S1. Association of social network measures with reduction in salt intake measured by 24hUNa in a random sample of 135 children [*]

Network measures	reduction in the amount of salt intake (95%CI), g/d †	*P* value
**Family support**		
Family member not supporting salt reduction (number) ‡		
>=3 (n=9)	-1.9 (-3.4 to -0.3)	0.018
2 (n=14)	-0.5 (-1.8 to 0.8)	0.466
1 (n=28)	-1.0 (-2.0 to -0.01)	0.049
0 (n=84)	Reference	-
Family member not supporting salt reduction (roles)		
Father (n=24)	-1.4 (-2.4 to -0.4)	0.004
Mother (n=9)	0.1 (-1.9 to 2.2)	0.89
Grandmother (n=16)	-1.3 (-2.9 to 0.4)	0.13
Grandparents (n=11)	-0.7 (-2.4 to 1.1)	0.42
None (n=84)	Reference	-
**Peer network measures**		
Network size	-0.01 (-0.05 to 0.03)	0.657
Network density	0.9 (-1.30 to 3.1)	0.410
Normalized degree centrality	-0.1 (-1.2 to 0.9)	0.825
**Teachers' engagement**		
Partial (n=56)	-0.6 (-1.4 to 0.2)	0.142
Full (n=79)	Reference	-
**Joint association**		
Family support (partial *versus* full)	-0.8 (-1.5 to -0.02)	0.046
Peer network (normalized degree centrality)	-0.5 (-1.3 to 0.4)	0.283
Teacher engagement (partial *versus* full)	-0.5 (-1.3 to 0.2)	0.172

*. 24hUNa=24-hour urine sodium excretion.
†. Reduction in salt intake indicates the change in salt intake from baseline to the end of trial (post-pre). The negative values reflect a smaller reduction in salt intake, after adjusting for age, sex, baseline body mass index and clustering effect of children nested within the same class in mixed linear models.
‡. *P* for trend=0.01. As an example, the interpretation of -1.87 (-3.41 to -0.32) for children who had ≥3 family members not supporting salt reduction, their reduction in salt intake (g/d) was 1.87g less per day, compared to their counterparts whose family supported salt reduction.
