# Supplementary material for: Social support, social network and salt-reduction behaviours in children: a substudy of the School-EduSalt trial
Source: BMJ Open. 2019 Jun 14;9(6):e028126. doi: 10.1136/bmjopen-2018-028126 (PMC6589018; doi:10.1136/bmjopen-2018-028126)
Supplement: Author's manuscript [file bmjopen-2018-028126.draft_revisions.pdf]

BMJ Open is committed to open peer review. As part of this commitment we make the peer review history of every article we publish publicly available.

When an article is published we post the peer reviewers' comments and the authors' responses online. We also post the versions of the paper that were used during peer review. These are the versions that the peer review comments apply to.

The versions of the paper that follow are the versions that were submitted during the peer review process. They are not the versions of record or the final published versions. They should not be cited or distributed as the published version of this manuscript.

BMJ Open is an open access journal and the full, final, typeset and author-corrected version of record of the manuscript is available on our site with no access controls, subscription charges or pay-per-view fees (http://bmjopen.bmj.com).

If you have any questions on BMJ Open's open peer review process please email info.bmjopen@bmj.com

BMJ Open

Social support, social network and salt-reduction behaviours in children: a sub-study of the School-EduSalt trial

Journal:	*BMJ Open*
Manuscript ID	bmjopen-2018-028126
Article Type:	Research
Date Submitted by the Author:	23-Nov-2018

Complete List of Authors:	Ma, Yuan; Peking University Health Science Centre, Department of Epidemiology and Biostatistics; Harvard University T H Chan School of Public Health, Department of Epidemiology<br>Feng, Xiangxian; Changzhi Medical College,<br>Ma, Jun; Institute of Child and Adolescent Health, Peking University Health Science Center,<br>He, Feng; Queen Mary University of London, Wolfson Institute of Preventive Medicine<br>Wang, Haijun<br>Zhang, Jing; The George Institute for Global Health at Peking University Health Science Center,<br>Xie , Wuxiang<br>Wu, Tao; The George Institute for Global Health at Peking University Health Science Center<br>Yin, Yunjian; Peking University School of Mathematical Sciences, Department of Probability and Statistics<br>Yuan, Jianhui; Changzhi Medical College,<br>MacGregor, Graham; Queen Mary University of London, Wolfson Institute of Preventive Medicine<br>Wu, Yangfeng; Peking University School of Public Health, Epidemiology

Keywords:	Hypertension < CARDIOLOGY, Social network, Social support, Behaviour change, Sodium consumption

SCHOLARONE™ Manuscripts

Social support, social network and salt-reduction behaviours in children: a sub-study of the School-EduSalt trial

Yuan Ma[1,2], Xiangxian Feng[3], Jun Ma[4], Feng J He[5], Haijun Wang[4], Jing Zhang[6], Wuxiang Xie[7], Tao Wu[6], Yunjian Yin[8], Jianhui Yuan[3], Graham MacGregor[5], Yangfeng Wu[1,6,7]

1. Department of Epidemiology and Biostatistics, Peking University School of Public Health, Beijing, China.
2. Department of Epidemiology, Harvard T.H. Chan School of Public Health, Boston, MA, USA.
3. Department of Preventive Medicine, Changzhi Medical College, Changzhi, China.
4. Institute of Child and Adolescent Health, Peking University Health Science Center, Beijing, China.
5. Wolfson Institute of Preventive Medicine, Barts and The London School of Medicine & Dentistry, Queen Mary University of London, London, UK.
6. The George Institute for Global Health, Peking University Health Science Center, Beijing, China.
7. Peking University Clinical Research Institute, Beijing, China.
8. Department of Probability and Statistics, Peking University School of Mathematical Sciences, Beijing, China.

Correspondence to: Dr. Yangfeng Wu, Department of Epidemiology and Biostatistics, Peking University School of Public Health, Beijing, 100088 China,

E-mail: wuyf@bjmu.edu.cn

Number of tables: 3

Number of figures: 3

Word counts excluding abstract and references: **3683**

For peer review only - http://bmjopen.bmj.com/site/about/guidelines.xhtml

ABSTRACT

Objectives Promoting positive behaviour changes such as reducing salt intake, is important to prevent lifestyle-related diseases, but is difficult to achieve. Social environment is proposed as a major challenge, with the underlying mechanisms being poorly understood. We determined whether social networks of children were associated with their behaviours to reduce salt intake.

Design A sub-study of a school-based behavioural intervention programme to reduce salt intake in children and their families (School-EduSalt), in which salt intake of children was significantly reduced by 25%.

Setting 14 primary schools in urban Changzhi, northern China.

Participants 603 children aged 10~12 years in the intervention arm.

Primary and Secondary Outcome Measures We developed a score assessing salt-reduction behaviours (SRB score) of children based on self-administered questionnaires. The SRB score was validated against the changes in salt intake measured by 24-hour urine collection in a random sample of 140 children, with one-unit increase in SRB score associated with a 0.31 gram/day greater reduction in salt intake (95%CI: 0.06~0.56, P=0.017).

Results The children from families where the father supported salt reduction had significantly higher mean SRB scores (All $P<0.05$). Children from a class with a smaller size and more friendship connectedness, as well as children having more friends in the class all showed higher SRB scores (All $P<0.05$). Children whose school teachers attended more frequently in the intervention program also had higher SRB scores ($P=0.043$).

Conclusion Social networks of children may influence their behaviours to reduce salt intake. Future salt-reduction programs may benefit from strategies that actively engage family members and school teachers, and strategies that enhance inter-connectivity among peers.

Key words: Salt reduction; Hypertension; Social networks; Social support; Behaviour change.

For peer review only - http://bmjopen.bmj.com/site/about/guidelines.xhtml

Strengths and Limitations

- Leveraging a randomised controlled trial that demonstrated a successful intervention strategy to reduce salt intake in children and their families, we identified the role of social network factors in driving favourable behaviours to reduce salt intake.
- The validity of the primary outcome was validated by the change in sodium intake measured by two-consecutive 24-hour urine collection.
- Given the exploratory nature of the study, data collected after the end of the main trial are subject to recall bias. Temporal order of the association warrants future study.
- The sensitivity analysis, using change in salt intake measured by 24h urinary sodium as the outcome, indicates the robustness of the major findings.

For peer review only

For peer review only - http://bmjopen.bmj.com/site/about/guidelines.xhtml

BACKGROUND

Cardiovascular disease (CVD) is a major cause of death globally despite substantial improvement in CVD treatment [1]. Dietary risk factors have become the largest contributors to global disease burden especially to cardiovascular diseases, with high sodium intake among the top ones [1 2]. Compelling evidence also highlights the crucial role of dietary and lifestyle-related behaviour change in curbing the global CVD pandemic [3-5]. Factors driving effective interventions to change behaviours are, however, unclear [1 6 7].

Abundant evidence from observational studies shows that social networks (i.e. social ties connected to the individual) may affect individuals' health behaviours such as obesity, smoking, fruit and vegetable consumption, partly through social support and social influence [8-10]. Evidence from interventional studies also demonstrates that intervention on tobacco control incorporating the role of network structure and social support is more effective than usual interventions [11-14]. Emerging evidence in children also emphasizes the role of peer influence in children's eating behaviours [15 16]. However, evidence examining role of social networks in behaviours to reduce salt intake is limited.

Salt intake is high in children in most populations [17-20], and it is shown that greater long-term health benefits can be achieved through a lower salt diet initiated early from childhood [21 22]. School-EduSalt trial is one of the few randomized trials worldwide that demonstrated a successful reduction of 25% in salt intake among children and families through a school-based education programme [20]. This study enabled us to examine the driving factors of effective behaviour changes to reduce salt intake in intervention settings. We conjectured that the social networks of children could play an important role. Therefore, the current study determined whether the three domains of children's social networks, including peer, family and teacher, were associated with the magnitude of salt reduction among children in the intervention group. Such knowledge could inform future salt reduction programmes and shed light on other lifestyle interventions.

For peer review only - http://bmjopen.bmj.com/site/about/guidelines.xhtml

METHODS

The School-EduSalt trial

School-EduSalt (**School** Based **Edu**cation Programme to Reduce **Salt**) was a cluster-randomized controlled trial carried out in 28 primary schools in an urban area of northern China. One class in grade 5 was selected from each school. The 28 classes were randomly allocated (1:1) to either intervention or control group. School-based salt-reduction education programme was delivered to all the children and their families in the 14 classes of the intervention group, while no specific intervention was delivered to the control. The duration of the intervention was a school semester (≈3.5 months). Around 10 children were randomly selected from each class and two adult family members of each of these children were invited to collect two-consecutive 24-hour urine at both baseline and end of trial. A detailed report of the study design [23] and the main results [20] had been published previously. Only brief methods related to this study were described here.

The children social networks study

The subjects of this study were all the schoolchildren aged 11.2±0.6 (SD) years in the intervention group. The primary outcome was the score on salt-reduction behaviours (hereinafter referred to as SRB score), validated by the reduction in salt intake measured by 24h urine sodium excretion before and after the intervention. Primary exposure measurements were factors related to the three major domains of the networks of children, including family support, peer network and teacher engagement. Children's peer networks were socio-centric networks with each of the 14 classes being a complete network, and all 603 children receiving intervention were recruited. For family and teacher network of the children, given data availability, we only assessed family support and teacher engagement.

Patient involvement

The school principals and head teachers of the included schools were involved in the study design stage for the development of study interventions and feasibility assessment of the study outcomes measurements of the main trial, from which the present study was further developed.

For peer review only - http://bmjopen.bmj.com/site/about/guidelines.xhtml

No participants were involved in setting the research question or the outcome measures, nor were they asked to advise on the interpretation or the writing up of the present study.

Data collection

Data used in this study were collected throughout the main trial, together with an additional post-trial survey specifically designed for this study (**Figure 1**). The post-trial survey was carried out among all the children in the intervention classes in the ninth month after the end of the intervention. Children were asked to fill out a self-administered questionnaire at school time for data on children's peer network and their recall of their family members' support for the School-EduSalt intervention program during the trial period. Virtually all children (603out of 604) completed the survey. In the main trial, two-consecutive 24-hour urine for each child, both before and after the intervention, was collected among a random sample of 141 children to estimate the change in salt intake [14,15]. More details are given below.

Outcome assessment

The SRB score was derived from three questions on behaviours related to salt reduction and collected in the post-trial survey. The three questions included the frequency of eating high-salt snacks (≥3 days/week, 1 to 3 days/week, almost never), the frequency of eating high-salt pickles (≥3 days/week, 1 to 3 days/week, almost never), and whether their families are taking actions to reduce salt intake (never, used to, now). Scores ranging from 1 to 3 were assigned to the answer of each question in order. The SRB score was calculated by summing up the standardized score (i.e. the z-score, in order to make the weight of each question comparable) from each question.

To validate SRB score, we assessed the association of the SRB score with the reduction in salt intake measured by 24h urine sodium excretion before and after the intervention among a random sample of children who made 24-hour urine collection as described above in the trial. Mixed linear models were used with school and class as random effects. The 24h urine collections deemed incomplete excluded from the analyses [20]. The number of children with both valid measurements of social network and completed 24-hour urine collection was 135.

For peer review only - http://bmjopen.bmj.com/site/about/guidelines.xhtml

Assessment of network measures

Family support for the salt reduction intervention from each family member was assessed by asking all children in the post-trial survey (i.e. supporting, opposing, neither supporting nor opposing). Children's peer network was defined as friends within the same class (boundary of the network). Each child (i.e. the nominator) was asked to nominate his/her friends (i.e. the nominees) from the same class who met at least two of the following conditions: (ⅰ). Play together almost every day during school time; (ⅱ). Play or do homework together frequently after school time (>3 times/week); (ⅲ). Often share snacks and toys; (ⅳ). Often share extracurricular books; (ⅴ). Go to school or go home after school together frequently. In order to take the strength of relation ties into account, we examined friendship ties with weighting adjustment using the number of above conditions they met. Peer network measures also included size and density of the networks as well as degree centrality and peer exposure of each child. By definition, size of network is the total number of children in each class. Density of network is the total number of friendship connections (ties) divided by the total possible number of ties within the same class and it reflects the overall interconnectedness of the children in the same class[24]. Degree centrality is the total number of friend ties directly connected to the child and it reflects the degree to which the child is connected to the network[24]. We used the normalized degree centrality that was divided by the size of class in order to make it comparable among classes. Both density and degree centrality reflect the connectivity of a network but from network and individual perspective respectively. Peer exposure (exposure to peers' salt-reduction behaviours) of a child was calculated by summing and averaging the SRB scores of his/her friends. Teacher engagement was recorded during the intervention and was defined as full (both the principle and headteacher attended all activities they had been invited to), partial (not all of them attend all the activities) and no (none attended the activities) engagement.

Data analysis

For peer review only - http://bmjopen.bmj.com/site/about/guidelines.xhtml

The association of family support with SRB score was tested using mixed linear models, which accounted for the clustering effect of children nested in the same class, with additional adjustment of age and gender.

For peer network measures, their association with SRB score was first quantified using mixed linear models. In the models, age and gender were adjusted. Peer networks of all children and their SRB scores were then visualized using network mapping techniques. Additionally, to examine whether children's SRB score was influenced by the SRB level of their friends (i.e. peer influence), analysis was performed from individual-focused and relation-focused facet respectively. Initially, we built traditional (individual-focused) mixed models on children's SRB score, with children's peer exposure, age and gender as explanatory variables. Although the dependence of the observations was considered in mixed models, this traditional method was still not perfect to address the dependence arising from the overlap of peer exposures. To address this issue and make inference from complex network structures, we then used exponential random graph models (ERGM) [25]. ERGM of social network structure was fit for each peer network based on the difference in SRB scores of each pair of friend ties to explain whether probability of the existence of friend ties between two children was associated with the similarity in SRB scores i.e. the so called homophily[26]. We applied the common model to all 14 classes to obtain estimates for each of the 14 classes. Finally, meta-analysis was used to pool the results across classes[27 28]. Fixed effect model would be used in absence of significant heterogeneity as assessed by I^2 statistics, otherwise random-effect model would be used.

Teacher engagement was examined using method similar to that for family support. Finally, we used mixed linear models to examine the overall effect of children's social networks by including the factors that best represent each network's influence on salt- reduction behaviours.

Sensitivity analysis

To test the robustness of the primary findings, we performed sensitivity analysis in a small random sample of children (n=135) with completed 24h urine collection at both baseline and the end of the trial. Specifically, change in salt intake (latter-former) assessed by 24-hour

For peer review only - http://bmjopen.bmj.com/site/about/guidelines.xhtml

urine sodium excretion, a well-accepted reliable method to assess salt intake, was used as the outcome. The association of three network components (i.e. peer, family and teacher network components) with change in salt intake of children were investigated using mixed models, consistent with primary analysis, with additionally adjustment for baseline salt intake.

Traditional data analysis was performed in SAS version 9.4. Network properties were calculated in Ucinet.6.0 [29] while networks were visualized in NetDraw version 2.155 [30]. ERGM models were fit using the *statnet* package in R environment [22].

For peer review only

For peer review only - http://bmjopen.bmj.com/site/about/guidelines.xhtml

RESULTS

Participant Characteristics

The average age of the 603 children (boys: 50.2 %) participating in the study was 11.2 (mean) ± 0.6 (SD) years old. Almost half (46%) gained full support from their families for the School-EduSalt salt-reduction program. The proportion of boys whose fathers didn't support the program was higher compared with girls (*P*=0.0035). Around 50% had teachers fully engaged in the intervention program (**Table 1**). The average size of the classes was 43 (range: 13-77). Salt-reduction behaviours of children are described in **Table 2**.

Validation of the Score on Salt-Reduction Behaviours (SRB scores)

In the random sample of 135 children with complete 24h urine collection both before and after intervention, the validation analysis showed that every 1 score increase in SRB score was associated with a 0.31g/d (95%CI: 0.06~0.56) greater reduction in salt intake (*P*=0.017). As further illustrated in **Figure 2**, the reduction in salt intake increased gradually from the lowest to the highest quartile of SRB score, presenting a significant linear trend (*P* trend =0.036), with a greater SRB score indicating a larger (favourable) reduction in salt intake.

SRB score and network measures

From the perspective of family network, with more family members not supporting salt reduction, the SRB score of children tended to decrease (*P* for trend<0.0001). The SRB score of children whose father didn't support salt reduction was significantly lower compared to the children whose father did (*P*<0.001). **(Table 3)**

From the perspective of the peer network, smaller size, higher density, and higher normalized degree centrality were significantly associated with a greater SRB score after adjusting for children's age and gender (**Table 3**). The visualization of children's peer network also provided complementary information supporting these quantitative results, with children of greater scores (i.e. greater nodes) tending to have denser local networks and the direct connections for smaller nodes tending to be sparser (**Figure 3**). For the role of peer exposure,

For peer review only - http://bmjopen.bmj.com/site/about/guidelines.xhtml

traditional analysis shows that every one-unit increase in the score of peer exposure was associated with a slight and non-significant increase of 0.07 (95%CI: -0.07 to 0.20, P=0.21) in the SRB score of children's (**Table 3**). Consistently, according to meta-analysis of the 14 exponential random graph models built for each of the 14 networks, similarity in SRB score was not found among friends (*P*>0.05). It shows that the likelihood of forming friend relation was only 2% higher for children whose SRB scores were closer (Odds Ratio = 1.02, 95%CI: 0.93 to 1.11, *P*=0.68, **Supplementary Figure S1**).

In terms of teacher engagement, the SRB scores for the 311 children in the schools that both the principal and head teacher fully participated in the intervention were significantly higher than those 292 children from the other schools, after adjusting for age and gender in the mixed model (Difference in SRB score: 0.33; 95%CI: 0.01 to 0.64, *P*=0.043, **Table 3**).

When network factors from the above three domains were examined together in the same model, the role of family support remained statistically significant, after adjusting for age, gender and clustering effect (*P*<0.0001). The association between SRB score and the degree of being connected to peers in the class as measured by the normalized degree centrality became borderline significant (*P*=0.13). Findings were similar for size and density of peer networks (data not shown). The association of SRB score with teacher engagement was also attenuated (**Table 3**).

In the final sensitivity analysis replacing SRB score with change in salt intake measured by 24-hour urinary sodium excretion as the outcome, results were consistent with primary findings. Specifically, with more family members not supporting salt reduction, the extent of the fall in salt intake of children tended to be less (*P* for trend=0.01), after adjusting for age, sex and baseline salt intake of children. Children whose father didn't support salt reduction also had an average of 1.39 gram (95%CI: 0.38-2.41) less reduction in daily salt intake compared to the children with support from all family member (P<0.001). The role of family support remained statistically significant after further accounting for factors of peer and teacher network. The association of change in salt intake with peer and teacher network

For peer review only - http://bmjopen.bmj.com/site/about/guidelines.xhtml

factors were not statistically significant in the smaller sample of 135 children, with point estimates showing essentially similar patterns. **(Supplementary Table S1)**

DISCUSSION

Our study is the first to explore the role of social networks in salt-reduction behaviours. The results demonstrated that salt-reduction behaviours in children were significantly influenced by their family support, peer networks and teacher engagement from multiple aspects. These findings offer new insights into the mechanisms that could explain the successful reduction of salt intake of children in the School EduSalt trial, which targeted the school class as a whole rather than individual child, and developed purposeful activities to engage families, teachers and school administrators. More importantly, the findings informed us on novel strategies for designing future salt reduction intervention programs through behaviour change. In a broader context, the exemplary findings from this study also shed light on the potential role of social networks in other diet and lifestyle interventions for the prevention and control of lifestyle-related diseases including, but not limited to, hypertension and cardiovascular diseases.

Our findings on the role of family networks in salt reduction interventions are consistent with the main body of evidence that demonstrates the role of family support in shaping smoking, physical activity and dietary behaviours [31-33]. Notably, besides the quantitative aspect of family support, our study further revealed that the fathers had more prominent influence on children's behaviour change related to salt reduction compared to other family members. This was, however, not surprising given the traditional Chinese culture where the father is usually the head of the family and could exert more influence on diet choices in the family. It suggests that identifying the head of network (opinion leader) and developing specific interventions targeting the head of network is important to enhance behaviour changes of the members within the network.

For peer networks, both density and degree centrality reflect a better interconnectivity of the network members but from network and individual perspective respectively. The networks that are better interconnected could provide children more opportunities to gain peer support [34] and had more power to influence their individual members, which would in turn lead to

For peer review only - http://bmjopen.bmj.com/site/about/guidelines.xhtml

better salt-reduction behaviour change. Likewise, children with more friends within one class may have a better sense of belonging to the class and were thus more likely to be involved in salt-reduction activities targeting the whole class. Similar inverse association between obesity and density of friend networks was also reported in an observational study previously [35]. By contrast, peer influence and homophily phenomena, which was reported for obesity [8] and smoking [9], were not found for salt reduction in our study. The results were consistent using both the traditional mixed model analysis and exponential random graph model analysis. This is likely due to the fact that children with obesity and smoking behaviours are likely to play together but children with the similar salt intake behaviour may not necessarily do. It indicates that, unlike smoking and obesity, salt reduction behaviours of a child may not influence its peer friendship, or vice versa.

As first revealed in our study, school teacher engagement in the intervention activity also significantly influenced the behaviours of salt reduction among children. Their role might be directly exerted through strengthening the children's attention to the intervention activities or their beliefs in the health messages delivered during the health education classes. It could also be mediated through their indirect influences on parents so that parents would pay more attention to the intervention program and hence provide more support to the children.

It was not until recently that the role of social networks in shaping individuals' attitude and behaviours in the domain of non-communicable diseases has been increasingly recognized [36]. Emerging intervention studies engaging different aspects of network components have successfully achieved desired behaviour changes [10-13]. As an example of employing the role of peer influence, observing videos of heroic peers eating fruit and vegetables was reported to increase fruit consumption of children [10]. It was also reported that intervention delivered by peers was more effective for smoking cessation compared to usual care [12]. Furthermore, modifying individual's network structures is also suggested to benefit social diffusion of desired behaviours [37]. For example, assigning intervention groups based on naturally-formed network, rather than random assignment, enhanced the effect of tobacco control among children [11]. Similarly, targeting nominated friends of individuals is also demonstrated to

For peer review only - http://bmjopen.bmj.com/site/about/guidelines.xhtml

increase adoption of a nutritional intervention [38]. Nevertheless, given the difficulty in implementing effective dietary and lifestyle intervention, the current research on incorporating individuals' social networks into nutrition and lifestyle intervention is far from sufficient. Since non-communicable diseases are rooted in unhealthy life styles that are expected to be changed mainly through behaviour changes. Our study, together with existing evidence[13], suggests the great potential to drive desired behaviour changes and improve public health by assessing social network context and engaging appropriate network interventions tailored to the nature of the behaviours to be targeted. Only when more powerful interventions changing the widely prevalent unhealthy life styles are developed would we prevent and control non-communicable diseases more effectively.

Our study has several limitations. First, our data on peer networks and family support was recalled after the end of the trial, which limited our ability to draw causal conclusion on the relationship of peer network factors with salt-reduction behaviours. Recall bias could also be an issue. Given that it is more likely that individuals misreported less favourable support as desired support rather than the other way around, thus blurring the difference we aimed to identify. The findings were likely to be more conservative. Second, given practical reasons, we only examined family support and teacher engagement for the social network factors from family and school perspective. Thirdly, for peer network, although the peer exposure defined in the current study was not associated with individuals' behaviours to reduce salt intake, other peer networks beyond the scope of the current study, such as friends out of the class and from the social media may play a role.

Our study also has several strengths. First, this is the first study to explore the role of social network factors in salt-reduction behaviours. Second, our study is based on a well-designed randomised controlled trial that successfully reduced salt intake in children and their families in the intervention arm by about one fourth in a school semester, which enabled us to address our research question and ensured the internal validity. Third, our study outcome, i.e. SRB score, was validated by the sodium intake estimated through 24h urine collection, which is the current gold method for measuring salt intake [39] in a random-selected sample of children.

For peer review only - http://bmjopen.bmj.com/site/about/guidelines.xhtml

Though a more ideal solution would be assessing behaviours using 24h urine collection for children, it is not feasible to implement this as it is costly, resource consuming, and challenging to ensure the completeness of 24h urine collection on a large scale.

CONCLUSION

Our study demonstrates that the social networks of children may play a role in shaping the behaviours to reduce salt intake in children. It provides new evidence for the benefit of integrating social network components into lifestyle interventions targeting behaviour changes. It has significant implication for future development of public health strategies for salt reduction as well as other lifestyle interventions.

Acknowledgements We thank the children and their families, the head teachers and school principals who participated in the study. We also thank all the team members from Changzhi Medical College, particularly Jianhui Yuan, Yanbo Han, Zhifang Li, Jianbin Zhang, Peifen Duan, Cailing Wei, Yanli Zhai, Ziqun Zhuo, Meng Xiao and Yunbin Zhang for their help with the data collection.

Contributors Yangfeng Wu and Yuan Ma designed this study. Yuan Ma developed the analysis plan. Yuan Ma, Yunjian Yin, Tao Wu and Wuxiang Xie performed statistical analyses. Yuan Ma and Yangfeng Wu wrote the manuscript. Yangfeng Wu, Feng J He, Graham A MacGregor, Xiangxian Feng, Jun Ma and Haijun Wang designed the main study (School-EduSalt trial). All authors have critically reviewed the manuscript and approved the final version.

Funding The study was funded by the UK Medical Research Council (MR/J015903/1).

Disclaimer The funder of the study had no role in the design of the study; the collection, analysis, and interpretation of the data; the writing of the manuscript; and the decision to submit the article for publication.

For peer review only - http://bmjopen.bmj.com/site/about/guidelines.xhtml

Participant consent Written informed consent was obtained from all the participants. For children, participant assent and parental written consent were obtained.

Competing interests All authors declare: no support from any organisation for the submitted work; no financial relationships with any organisations that might have an interest in the submitted work in the previous three years.

Ethics approval This study was approved by Peking University Health Science Centre Institutional Review Board (IRB00001052-12072) and Queen Mary University of London Research Ethics Committee (QMREC2012/81).

Data sharing statement The datasets used during the current study are available from the corresponding author on reasonable request.

REFERENCES

1. GBD Risk Factors Collaborators. Global, regional, and national comparative risk assessment of 79 behavioural, environmental and occupational, and metabolic risks or clusters of risks in 188 countries, 1990-2013: a systematic analysis for the Global Burden of Disease Study 2013. *Lancet* 2015;386(10010):2287-323. doi: 10.1016/S0140-6736(15)00128-2.
2. Mozaffarian D, Wilson PWF, Kannel WB. Beyond Established and Novel Risk Factors: Lifestyle Risk Factors for Cardiovascular Disease. *Circulation* 2008;117(23):3031-38. doi: 10.1161/circulationaha.107.738732.
3. Eckel RH, Jakicic JM, Ard JD, et al. 2013 AHA/ACC guideline on lifestyle management to reduce cardiovascular risk: a report of the American College of Cardiology/American Heart Association Task Force on Practice Guidelines. *Journal of the American College of Cardiology* 2014;63(25 Pt B):2960-84. doi: 10.1016/j.jacc.2013.11.003 [published Online First: 2013/11/19].
4. Wu Y, Benjamin EJ, MacMahon S. Prevention and Control of Cardiovascular Disease in the Rapidly Changing Economy of China. *Circulation* 2016;133(24):2545-60. doi: 10.1161/circulationaha.115.008728 [published Online First: 2016/06/15].
5. Amine E, Baba N, Belhadj M, et al. Diet, nutrition and the prevention of chronic diseases: report of a Joint WHO/FAO Expert Consultation: World Health Organization 2002.
6. Knapper JT, Ghasemzadeh N, Khayata M, et al. Time to Change Our Focus: Defining, Promoting, and Impacting Cardiovascular Population Health. *Journal of the American College of Cardiology* 2015;66(8):960-71. doi: 10.1016/j.jacc.2015.07.008 [published Online First: 2015/08/22].

For peer review only - http://bmjopen.bmj.com/site/about/guidelines.xhtml

7. Newson RS, Elmadfa I, Biro G, et al. Barriers for progress in salt reduction in the general population. An international study. *Appetite* 2013;71:22-31. doi: 10.1016/j.appet.2013.07.003.
8. Christakis NA, Fowler JH. The Spread of Obesity in a Large Social Network over 32 Years. *New England Journal of Medicine* 2007;357(4):370-79.
9. Christakis NA, Fowler JH. The Collective Dynamics of Smoking in a Large Social Network. *New England Journal of Medicine* 2008;358(21):2249-58.
10. Lowe CF, Horne PJ, Tapper K, et al. Effects of a peer modelling and rewards-based intervention to increase fruit and vegetable consumption in children. *European journal of clinical nutrition* 2004;58(3):510-22.
11. Valente TW, Hoffman BR, Ritt-Olson A, et al. Effects of a social-network method for group assignment strategies on peer-led tobacco prevention programs in schools. *Am J Public Health* 2003;93(11):1837-43.
12. Malchodi CS, Oncken C, Dornelas EA, et al. The effects of peer counseling on smoking cessation and reduction. *Obstet Gynecol* 2003;101(3):504-10.
13. Valente TW. Network interventions. *Science* 2012;337(6090):49-53. doi: 10.1126/science.1217330 [published Online First: 2012/07/07].
14. Campbell R, Starkey F, Holliday J, et al. An informal school-based peer-led intervention for smoking prevention in adolescence (ASSIST): a cluster randomised trial. *Lancet* 2008;371(9624):1595-602. doi: 10.1016/s0140-6736(08)60692-3 [published Online First: 2008/05/13].
15. Fletcher A, Bonell C, Sorhaindo A. You are what your friends eat: systematic review of social network analyses of young people's eating behaviours and bodyweight. *J Epidemiol Community Health* 2011;65(6):548-55. doi: 10.1136/jech.2010.113936 [published Online First: 2011/03/24].
16. Buller DB, Morrill C, Taren D, et al. Randomized trial testing the effect of peer education at increasing fruit and vegetable intake. *J Natl Cancer Inst* 1999;91(17):1491-500. [published Online First: 1999/09/02].
17. Marrero NM, He FJ, Whincup P, et al. Salt intake of children and adolescents in South london: consumption levels and dietary sources. *Hypertension* 2014;63(5):1026-32. doi: 10.1161/hypertensionaha.113.02264.
18. Grimes CA, Riddell LJ, Campbell KJ, et al. Dietary salt intake assessed by 24 h urinary sodium excretion in Australian schoolchildren aged 5-13 years. *Public Health Nutr* 2013;16(10):1789-95. doi: 10.1017/s1368980012003679.
19. Morinaga Y, Tsuchihashi T, Ohta Y, et al. Salt intake in 3-year-old Japanese children. *Hypertension research : official journal of the Japanese Society of Hypertension* 2011;34(7):836-9. doi: 10.1038/hr.2011.55 [published Online First: 2011/05/20].
20. FJ H, Y W, FengXX, et al. School based education programme to reduce salt intake in children and their families (School-EduSalt): cluster randomised controlled trial. . *BMJ* 2015;350:h770.
21. Cooper R, Soltero I, Liu K, et al. The association between urinary sodium excretion and blood pressure in children. *Circulation* 1980;62(1):97-104. [published Online First: 1980/07/01].

For peer review only - http://bmjopen.bmj.com/site/about/guidelines.xhtml

22. Hofman A, Hazebroek A, Valkenburg HA. A randomized trial of sodium intake and blood pressure in newborn infants. *JAMA* 1983;250(3):370-3. [published Online First: 1983/07/15].
23. He FJ, Wu Y, Ma J, et al. A school-based education programme to reduce salt intake in children and their families (School-EduSalt): protocol of a cluster randomised controlled trial. *BMJ open* 2013;3(7):e003388-e88. doi: 10.1136/bmjopen-2013-003388.
24. Hawe P, Webster C, Shiell A. A glossary of terms for navigating the field of social network analysis. *J Epidemiol Community Health* 2004;58(12):971-5.
25. Goodreau SM. Advances in Exponential Random Graph (p*) Models Applied to a Large Social Network. *Social networks* 2007;29(2):231-48. doi: 10.1016/j.socnet.2006.08.001 [published Online First: 2008/05/02].
26. McPherson M, Smith-Lovin L, Cook JM. Birds of a feather: Homophily in social networks. *Annual Review of Sociology* 2001:415-44.
27. Snijders TAB, Baerveldt C. A multilevel network study of the effects of delinquent behavior on friendship evolution. *The Journal of Mathematical Sociology* 2003;27(2-3):123-51. doi: 10.1080/00222500305892.
28. Valente TW, Fujimoto K, Chou C-P, et al. Adolescent Affiliations and Adiposity: A Social Network Analysis of Friendships and Obesity. *Journal of Adolescent Health* 2009;45(2):202-04.
29. Borgatti SP, Everett MG, Freeman LC. Ucinet 6 for Windows: Software for Social Network Analysis: Analytic Technologies 2002.
30. Borgatti, S.P. 2002. NetDraw: Graph Visualization Software. Harvard: Analytic Technologies.
31. Berkman LF, Glass T, Brissette I, et al. From social integration to health: Durkheim in the new millennium. *Soc Sci Med* 2000;51(6):843-57.
32. Scholz U, Ochsner S, Hornung R, et al. Does social support really help to eat a low-fat diet? Main effects and gender differences of received social support within the Health Action Process Approach. *Applied psychology Health and well-being* 2013;5(2):270-90. doi: 10.1111/aphw.12010 [published Online First: 2013/04/30].
33. Niermann CY, Kremers SP, Renner B, et al. Family Health Climate and Adolescents' Physical Activity and Healthy Eating: A Cross-Sectional Study with Mother-Father-Adolescent Triads. *PloS one* 2015;10(11):e0143599. doi: 10.1371/journal.pone.0143599 [published Online First: 2015/11/26].
34. Gesell SB, Barkin SL, Valente TW. Social network diagnostics: a tool for monitoring group interventions. *Implement Sci* 2013;8:116.
35. Lee WJ, Youm Y, Rhee Y, et al. Social network characteristics and body mass index in an elderly Korean population. *Journal of preventive medicine and public health = Yebang Uihakhoe chi* 2013;46(6):336-45. doi: 10.3961/jpmph.2013.46.6.336 [published Online First: 2013/12/19].
36. Luke DA, Harris JK. Network analysis in public health: history, methods, and applications. *Annu Rev Public Health* 2007;28:69-93.
37. Laranjo L, Arguel A, Neves AL, et al. The influence of social networking sites on health behavior change: a systematic review and meta-analysis. *Journal of the American Medical Informatics Association* 2015;22(1):243-56. doi: 10.1136/amiajnl-2014-002841.

For peer review only - http://bmjopen.bmj.com/site/about/guidelines.xhtml

For peer review only

38. Kim DA, Hwong AR, Stafford D, et al. Social network targeting to maximise population behaviour change: a cluster randomised controlled trial. *The Lancet* 2015;386(9989):145-53. doi: https://doi.org/10.1016/S0140-6736(15)60095-2.

39. McLean RM. Measuring Population Sodium Intake: A Review of Methods. *Nutrients* 2014;6(11):4651-62. doi: 10.3390/nu6114651.

For peer review only

For peer review only - http://bmjopen.bmj.com/site/about/guidelines.xhtml

Table 1. Participant characteristics

Characteristics	All (N=603)
Age (years)	11.2±0.6
Boy, n (%)	303 (50.2)
**Family support,** n (%)	
Family members not supporting salt reduction (number)	
>=3	72 (12.0)
2	91 (15.1)
1	160 (26.6)
0	279 (46.3)
Family members not supporting salt reduction (roles)	
None	279 (46.3)
Father	154 (25.6)
Others *	169 (28.1)
**Peer network measures, n (%)**	
Network size (number of children within the class) †	43±16
Network density (in %) †	11±7
Number of friends	4 (2-5)
Normalized degree centrality (in %) ‡	24 (12-43)
Peer exposure (score on salt-reduction behaviors)	0.04 (-0.70-0.80)
**Teacher engagement, n (%)**	
Full	311 (51.6)
Partial	292 (48.4)

Data are shown in the format of mean ± SD, median (P25 - P75), and n (%).
*The mother or grandparents.
†Measures at cluster (class) level. Density of network is the total number of friendship connections (ties) divided by the total possible number of friendship ties within the same class.
‡Normalized degree centrality is the number of friends within the class divided by the size of class.

For peer review only - http://bmjopen.bmj.com/site/about/guidelines.xhtml

Table 2. Description of children's salt-reduction behaviors

Questions related to salt reduction (score assigned for each answer)	All (N=603)
**Q1.Frequency of eating pickles, N(%)**	
Almost never (=3)	446 (74.0)
1~3days/week (=2)	147 (24.3)
≥3 days/week(=1)	10 (1.7)
**Q2.Frequency of eating high-salt snacks, N(%)**	
Almost never (=3)	166 (27.6)
1~3days/week (=2)	358 (59.3)
≥3 days/week(=1)	78 (13.0)
**Q3.Family's action on salt reduction, N(%)**	
Have been reducing salt (=3)	401 (66.6)
Used to (=2)	172 (28.6)
Never (=1)	29 (4.8)
**Sum of Q1-Q3 scores, N(%)**	
7-9	95 (15.8)
4-6	482 (80.2)
0-3	24 (4.0)
**SRB score (the sum of standardized scores), (Mean ± SD)**	0±1.9

For peer review only

For peer review only - http://bmjopen.bmj.com/site/about/guidelines.xhtml

Table 3 Association of SRB score with social network measures among all 603 children

Network measures	Regression coefficient (95%CI) *	*P* value
**Family support**		
Family member not supporting salt reduction (number) †		
>=3	-1.1 (-1.6 to -0.6)	<0.0001
2	-1.0 (-1.4 to -0.5)	<0.0001
1	-0.6 (-0.9 to -0.2)	0.003
0	Reference	-
Family member not supporting salt reduction (roles)		
Father	-1.3 (-1.6 to -0.9)	<0.0001
Others	-0.6 (-1.0 to -0.2)	0.002
None	Reference	-
**Peer network measures**		
Network size (number of children within the class)	-0.01 (-0.02 to -0.002)	0.026
Network density (in %)	0.94 (0.06 to 1.82)	0.036
Normalized degree centrality (in %)	0.5 (0.01 to 0.99)	0.044
Peer exposure (score on salt-reduction behaviors)	0.07 (-0.07 to 0.20)	0.205
**Teachers' engagement**		
Partial	-0.33 (-0.64 to -0.01)	0.043
Full	Reference	-
**Joint association**		
Family support (partial versus full)	-0.82 (-1.13 to -0.52)	<0.0001
Peer network (normalized degree centrality)	0.37 (-0.11 to 0.85)	0.13
Teacher engagement (partial versus full)	-0.25 (-0.56 to 0.07)	0.20

*Linear regression coefficients for corresponding network measures in the mixed models on SRB score.
†*P* for trend<0.0001.

For peer review only - http://bmjopen.bmj.com/site/about/guidelines.xhtml

Figure legends

Figure 1. School-EduSalt network study design.

Figure 2. Change in salt intake measured by 24h urinary sodium across the quantiles of SRB score in children.

Figure 3. Visualization of peer networks in the 14 intervention schools.

Supplementary Figure S1. The association between the existence of friend ties and similarity in SRB score.

For peer review only

For peer review only - http://bmjopen.bmj.com/site/about/guidelines.xhtml

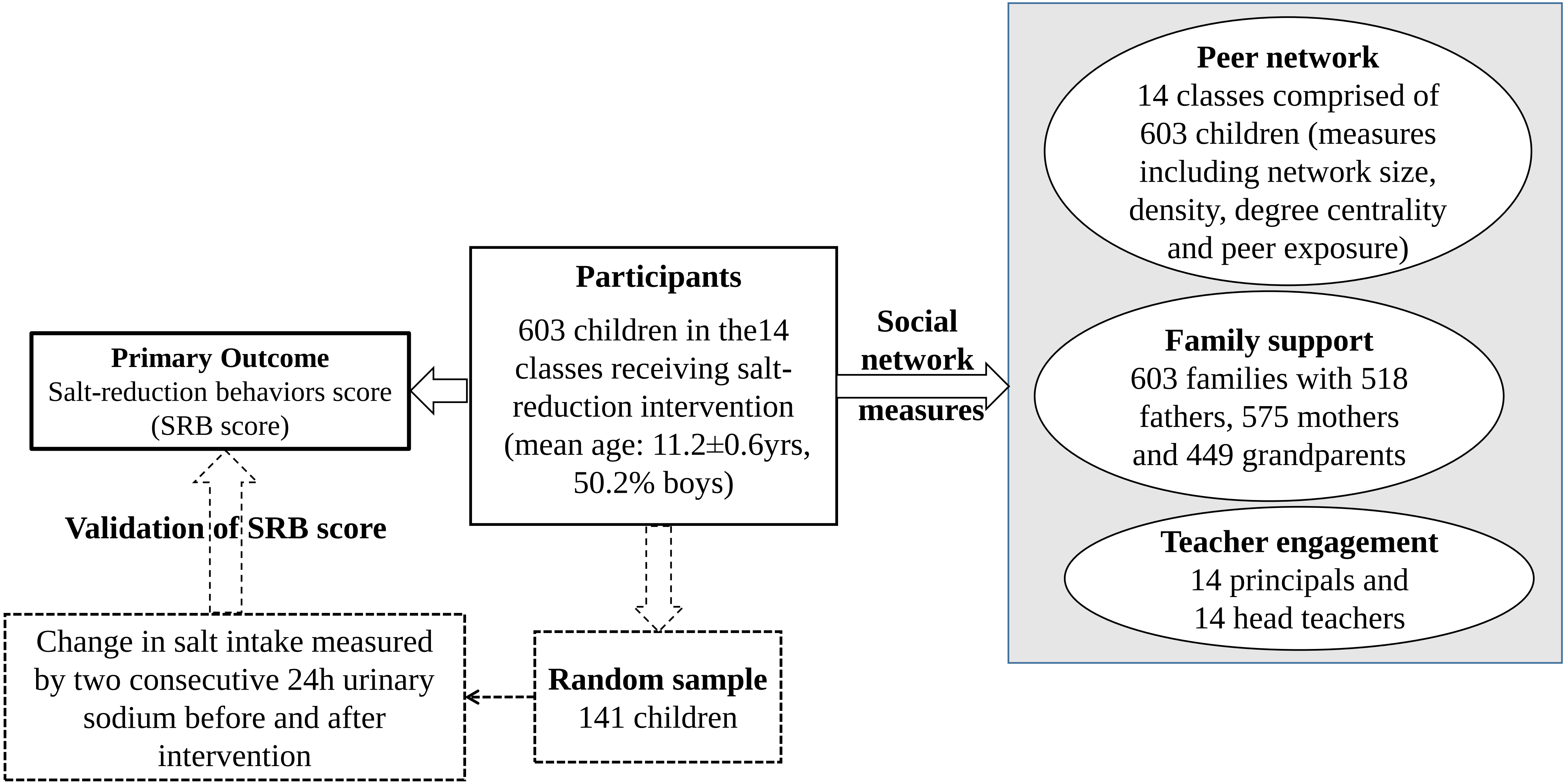

Figure 1 Study design

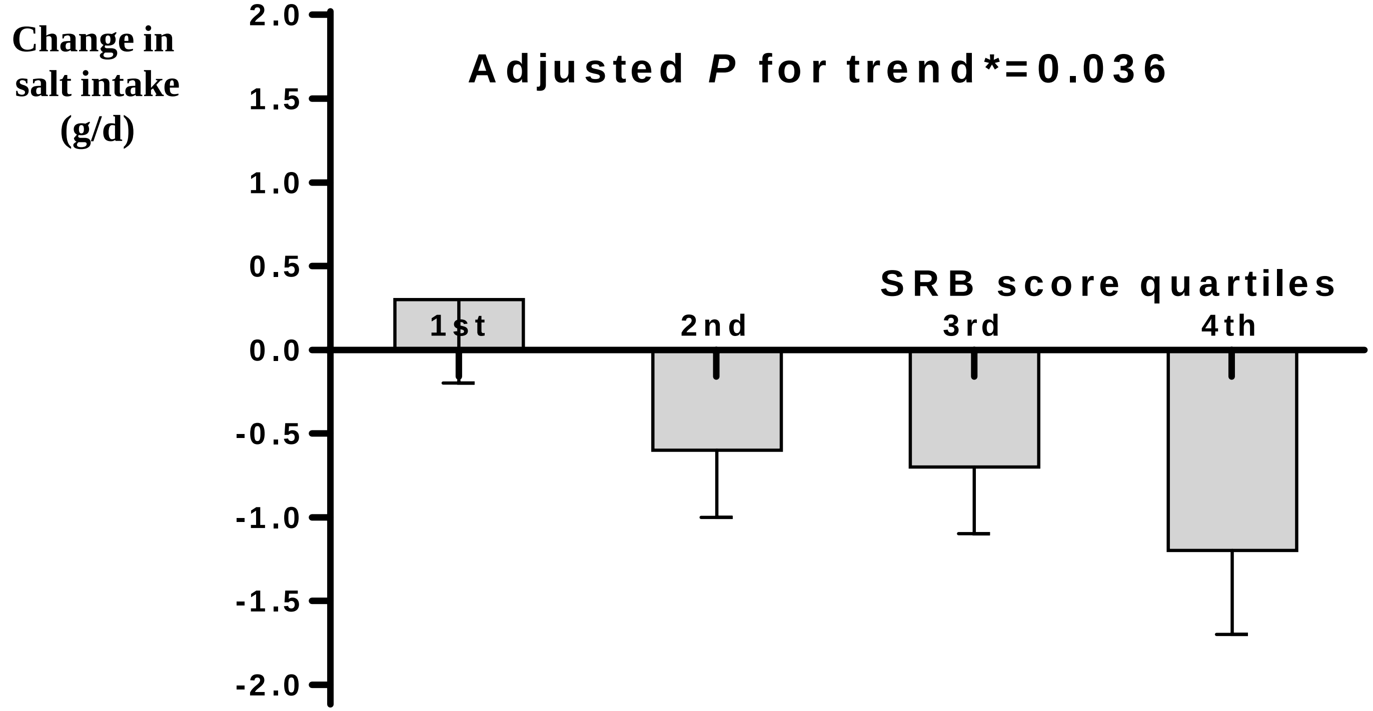

Figure 2. Change in salt intake measured by 24-hour urinary sodium excretion across the quartiles of the score on salt –reduction behaviors (SRB score).

*Adjusting for age, sex and body mass index.

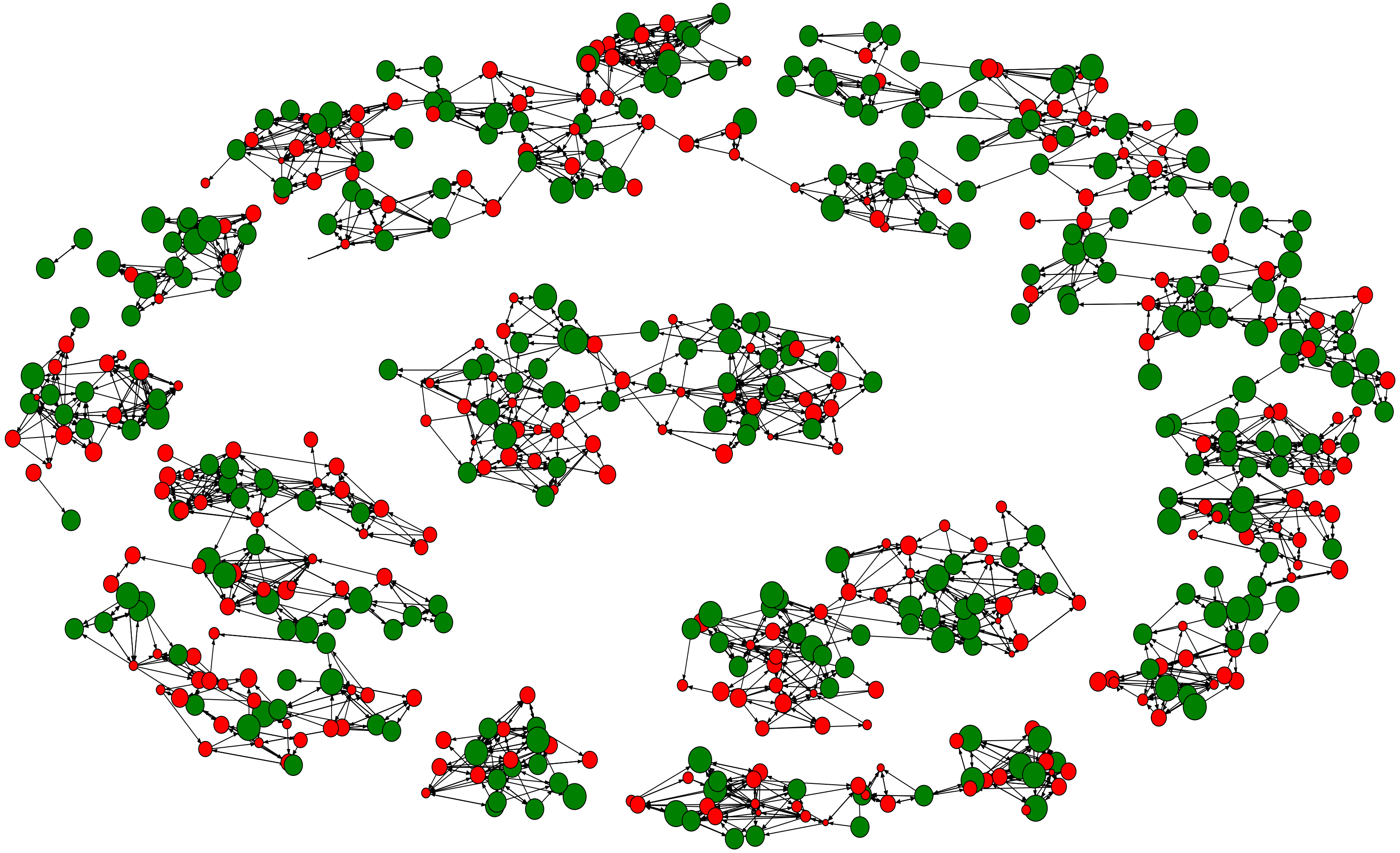

Figure 3. Visualization of peer networks in the 14 intervention schools

*Each node represents a child. The tie between two nodes denotes a friend relation. The size of each node is proportional to the score on salt-reduction behaviour (A higher score indicates a greater reduction in salt intake). Nodes with score above the median were painted in green colour and those below the median were in red.

Online Supplementary Material

Figure S1. The association between the existence of friend ties and similarity in SRB score

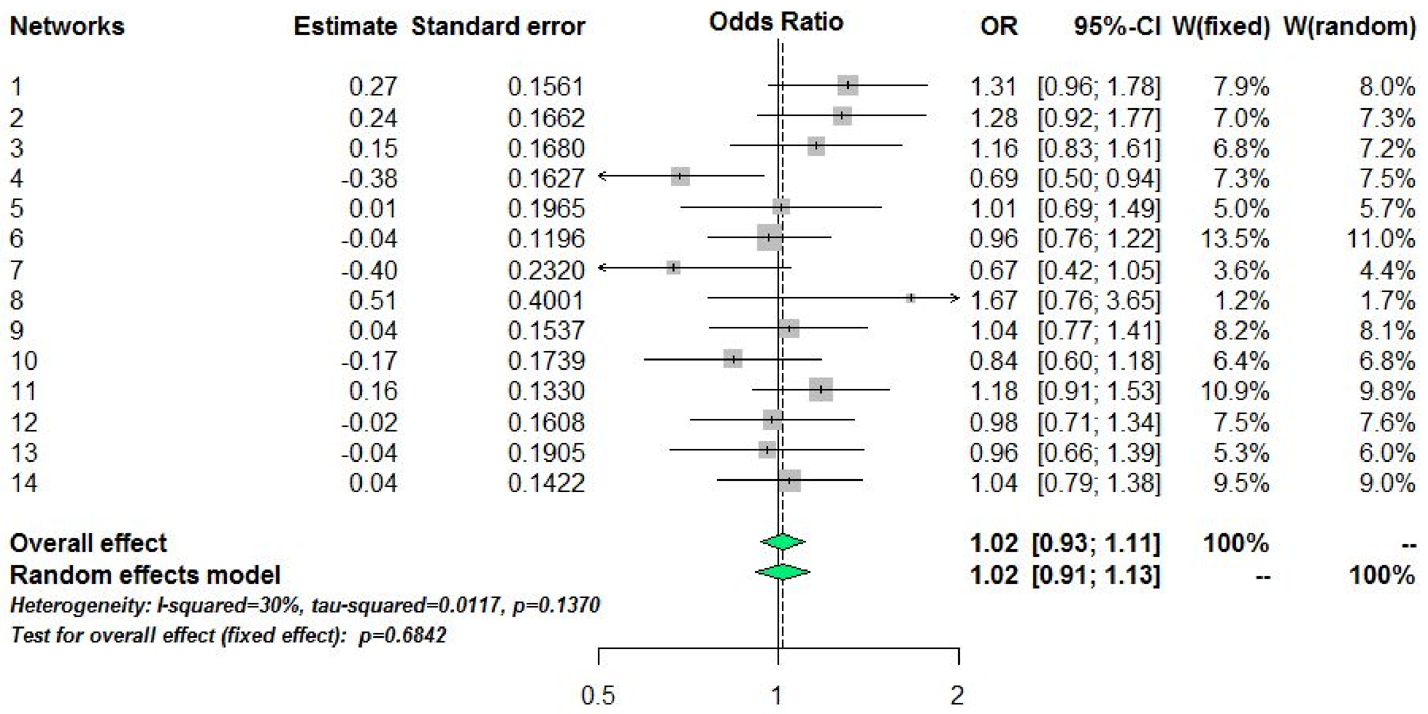

*Odds ratio of the existence of a friend tie associated with one-unit increase in the similarity of SRB score between each pair of children.

Supplementary Table S1. Association of social network measures with changes in salt intake measured by 24hUNa in a random sample of 135 children *

Network measures	Adjusted difference in the change in salt intake (95%CI), g/d †	*P* value
**Family support**		
Family member not supporting salt reduction (number) ‡		
>=3 (n=9)	1.87 (0.32 to 3.41)	0.0184
2 (n=14)	0.48 (-0.82 to 1.79)	0.4661
1 (n=28)	0.98 (0.01 to 1.95)	0.0486
0 (n=84)	Reference	-
Family member not supporting salt reduction (roles)		
The Father (n=24)	1.39 (0.38 to 2.41)	0.008
Others (n=27)	0.64 (-0.37 to 1.65)	0.211
None (n=84)	Reference	-
**Peer network measures**		
Network size	0.01 (-0.03 to 0.05)	0.657
Network density	-0.92 (-3.13 to 1.29)	0.410
Normalized degree centrality	0.12 (-0.93 to 1.16)	0.825
**Teachers' engagement**		
Partial (n=56)	0.59 (-0.20 to 1.38)	0.142
Full (n=79)	Reference	-
**Joint association**		
Family support (partial *versus* full)	0.77 (0.02 to 1.52)	0.046
Peer network (normalized degree centrality)	0.46 (-0.39 to 1.32)	0.283
Teacher engagement (partial *versus* full)	0.53 (-0.24 to 1.30)	0.172

*. 24hUNa=24-hour urine sodium excretion.
†. Change in salt intake indicates the change from baseline to the end of trial (post-pre). For the difference in the change in salt intake, negative values reflect larger reduction in salt intake. The adjusted differences correspond to the difference from mixed models after adjusting for age, sex baseline salt intake and clustering effect of children nested within the same class.
‡. *P* for trend=0.01.

For peer review only

STROBE Statement—Checklist of items that should be included in reports of ***cross-sectional studies***

	Item No	Recommendation
**Title and abstract (√, page1) (√, page 2)**	1	(*a*) Indicate the study's design with a commonly used term in the title or the abstract
		(*b*) Provide in the abstract an informative and balanced summary of what was done and what was found
**Introduction**		
Background/rationale **(√, page 4)**	2	Explain the scientific background and rationale for the investigation being reported
Objectives **(√, page 4)**	3	State specific objectives, including any prespecified hypotheses
**Methods**		
Study design **(√, page 5)**	4	Present key elements of study design early in the paper
Setting **(√, page 5-6)**	5	Describe the setting, locations, and relevant dates, including periods of recruitment, exposure, follow-up, and data collection
Participants **(√, page 5 and figure 1)**	6	(*a*) Give the eligibility criteria, and the sources and methods of selection of participants
Variables **(√, page 6-7)**	7	Clearly define all outcomes, exposures, predictors, potential confounders, and effect modifiers. Give diagnostic criteria, if applicable
Data sources/ measurement **(√, page 5-7)**	8*	For each variable of interest, give sources of data and details of methods of assessment (measurement). Describe comparability of assessment methods if there is more than one group
Bias **(√, page 8)**	5	Describe any efforts to address potential sources of bias
Study size **(NA)**	10	Explain how the study size was arrived at
Quantitative variables **(√, page 7-9)**	11	Explain how quantitative variables were handled in the analyses. If applicable, describe which groupings were chosen and why
Statistical methods **(√, page 7-9)**	12	(*a*) Describe all statistical methods, including those used to control for confounding
**(Page 10, subgroup analysis)**		(*b*) Describe any methods used to examine subgroups and interactions
		(*c*) Explain how missing data were addressed
**(NA)**		(*d*) If applicable, describe analytical methods taking account of sampling strategy
**(√, Page 9)**		(*e*) Describe any sensitivity analyses
**Results**		
Participants **(√, page 5, page 10 and figure 1)**	13*	(a) Report numbers of individuals at each stage of study—eg numbers potentially eligible, examined for eligibility, confirmed eligible, included in the study, completing follow-up, and analysed
		(b) Give reasons for non-participation at each stage
		(c) Consider use of a flow diagram
Descriptive data **(√, page 8， figure 1 and table 1)**	14*	(a) Give characteristics of study participants (eg demographic, clinical, social) and information on exposures and potential confounders
**(Figure 1)**		(b) Indicate number of participants with missing data for each variable of interest

For peer review only - http://bmjopen.bmj.com/site/about/guidelines.xhtml

Outcome data **(NA)**	15*	Report numbers of outcome events or summary measures
Main results **(√)** **(√, table 1~3)**	16	(*a*) Give unadjusted estimates and, if applicable, confounder-adjusted estimates and their precision (eg, 95% confidence interval). Make clear which confounders were adjusted for and why they were included
		(*b*) Report category boundaries when continuous variables were categorized
**(NA)**		(*c*) If relevant, consider translating estimates of relative risk into absolute risk for a meaningful time period
Other analyses **(Page11, table S1)**	17	Report other analyses done—eg analyses of subgroups and interactions, and sensitivity analyses
**Discussion**		
Key results **(√, page 12)**	18	Summarise key results with reference to study objectives
Limitations **(√, page 14)**	19	Discuss limitations of the study, taking into account sources of potential bias or imprecision. Discuss both direction and magnitude of any potential bias
Interpretation **(√, page 11-14)**	20	Give a cautious overall interpretation of results considering objectives, limitations, multiplicity of analyses, results from similar studies, and other relevant evidence
Generalisability **(√, page 14)**	21	Discuss the generalisability (external validity) of the study results
**Other information**		
Funding **(√, page 15)**	22	Give the source of funding and the role of the funders for the present study and, if applicable, for the original study on which the present article is based

*Give information separately for exposed and unexposed groups.

Note: An Explanation and Elaboration article discusses each checklist item and gives methodological background and published examples of transparent reporting. The STROBE checklist is best used in conjunction with this article (freely available on the Web sites of PLoS Medicine at http://www.plosmedicine.org/, Annals of Internal Medicine at http://www.annals.org/, and Epidemiology at http://www.epidem.com/). Information on the STROBE Initiative is available at www.strobe-statement.org.

For peer review only

BMJ Open

Social support, social network and salt-reduction behaviours in children: a sub-study of the School-EduSalt trial

Journal:	*BMJ Open*
Manuscript ID	bmjopen-2018-028126.R1
Article Type:	Research
Date Submitted by the Author:	23-Apr-2019
Complete List of Authors:	Ma, Yuan; Peking University Health Science Centre, Department of Epidemiology and Biostatistics; Harvard University T H Chan School of Public Health, Department of Epidemiology<br>Feng, Xiangxian; Changzhi Medical College,<br>Ma, Jun; Institute of Child and Adolescent Health, Peking University Health Science Center,<br>He, Feng; Queen Mary University of London, Wolfson Institute of Preventive Medicine<br>Wang, Haijun<br>Zhang, Jing; The George Institute for Global Health at Peking University Health Science Center,<br>Xie , Wuxiang<br>Wu, Tao; The George Institute for Global Health at Peking University Health Science Center<br>Yin, Yunjian; Peking University School of Mathematical Sciences, Department of Probability and Statistics<br>Yuan, Jianhui; Changzhi Medical College,<br>MacGregor, Graham; Queen Mary University of London, Wolfson Institute of Preventive Medicine<br>Wu, Yangfeng; Peking University School of Public Health, Epidemiology
**Primary Subject Heading**:	Public health
Secondary Subject Heading:	Epidemiology, Evidence based practice, Health policy, Nutrition and metabolism
Keywords:	Hypertension < CARDIOLOGY, Social network, Social support, Behaviour change, Sodium consumption

SCHOLARONE™
Manuscripts

Social support, social network and salt-reduction behaviours in children: a sub-study of the School-EduSalt trial

Yuan Ma[1, 2], Xiangxian Feng[3], Jun Ma[4], Feng J He[5], Haijun Wang[4], Jing Zhang[6], Wuxiang Xie[7], Tao Wu[6], Yunjian Yin[8], Jianhui Yuan[3], Graham MacGregor[5], Yangfeng Wu[1,6, 7]

1. Department of Epidemiology and Biostatistics, Peking University School of Public Health, Beijing, China.
2. Department of Epidemiology, Harvard T.H. Chan School of Public Health, Boston, MA, USA.
3. Department of Preventive Medicine, Changzhi Medical College, Changzhi, China.
4. Institute of Child and Adolescent Health, Peking University Health Science Center, Beijing, China.
5. Wolfson Institute of Preventive Medicine, Barts and The London School of Medicine & Dentistry, Queen Mary University of London, London, UK.
6. The George Institute for Global Health, Peking University Health Science Center, Beijing, China.
7. Peking University Clinical Research Institute, Beijing, China.
8. Department of Probability and Statistics, Peking University School of Mathematical Sciences, Beijing, China.

Correspondence to: Dr. Yangfeng Wu, Department of Epidemiology and Biostatistics, Peking University School of Public Health, Beijing, 100088 China,

E-mail: wuyf@bjmu.edu.cn

Number of tables: 3

Number of figures: 3

Word counts: **3403**

For peer review only

For peer review only - http://bmjopen.bmj.com/site/about/guidelines.xhtml

ABSTRACT

Objectives Healthy behaviour changes such as reducing salt intake, are important to prevent lifestyle-related diseases. Social environment is a major challenge to achieve such behaviours, but the explicit mechanisms remain largely unknown. We investigated whether social networks of children were associated with their behaviours to reduce salt intake.

Design An ancillary study of a school-based cluster randomized controlled trial to reduce salt intake in children and their families (School-EduSalt), in which salt intake of children was significantly reduced by 25%.

Setting 14 primary schools in urban Changzhi, northern China.

Participants 603 children aged 10-12 years in the intervention arm.

Primary and Secondary Outcome Measures We developed a score assessing salt-reduction behaviours (SRB score) of children based on self-administered questionnaires. The SRB score was validated by the changes in salt intake measured by 24-hour urine collection in a random sample of 135 children. A one-unit increase in SRB score was associated with a 0.31 gram/day greater reduction in salt intake during the trial (95%CI: 0.06 to 0.57, P=0.016).

Results Children from families with more family members not supporting salt reduction had significantly lower SRB scores (P<0.0001). Children from a class with a smaller size and from a class with more friendship connections, as well as children having more friends within the class all showed higher SRB scores (All P <0.05). Children whose school teachers attended the intervention program more frequently also had higher SRB scores (P=0.043).

Conclusion Social networks were associated with the behaviours to reduce salt intake in children. Future salt-reduction programs may benefit from strategies that actively engage families and teachers, and strategies that enhance inter-connectivity among peers.

Key words: Salt reduction; Hypertension; Social networks; Social support; Behaviour change.

For peer review only - http://bmjopen.bmj.com/site/about/guidelines.xhtml

Strengths and Limitations

- Leveraging a randomised controlled trial that demonstrated a successful intervention strategy to reduce salt intake in children and their families, we identified the social network factors associated with behaviours to reduce salt intake in children.
- The validity of the primary outcome was validated by the change in sodium intake measured by 24-hour urine collections.
- Advanced social network analysis techniques in addition to the traditional methods were used to carefully examine the role of peer influence in salt reduction.
- The sensitivity analysis, using change in salt intake measured by 24h urinary sodium as the outcome, showed consistent results, suggesting the robustness of the major findings.
- Given the exploratory nature of the study, data collected after the end of the main trial were subject to recall bias. Temporal order of the association warrants future study.

For peer review only

For peer review only - http://bmjopen.bmj.com/site/about/guidelines.xhtml

BACKGROUND

Cardiovascular disease (CVD) is a major cause of death globally despite substantial improvement in CVD treatment.[1] Dietary risk factors are the largest contributors to global disease burden, with high sodium intake among the top ones.[1] Compelling evidence also highlights the crucial role of dietary and lifestyle-related behaviour changes in curbing global CVD pandemic, [2-4] but factors driving effective behavioural interventions are not clear. [5-7]

Evidence from observational studies suggests that social networks (i.e. social ties connected to the individual) may affect individuals' health behaviours such as obesity, smoking, fruit and vegetable consumption, partly through social support and social influence. [8-10] Data from interventional studies also demonstrate that intervention on tobacco control incorporating the role of network structure and social support is more effective than usual interventions. [11-14] Emerging evidence in children also emphasizes the role of peer influence in children's eating behaviours.[15 16] However, evidence examining role of social networks in behaviours to reduce salt intake is lacking.

Salt intake is high in children in most countries,[17-20] and it is shown that greater long-term health benefits can be achieved through a lower salt diet initiated early from childhood.[21 22] School-EduSalt trial is one of the few randomized trials worldwide that demonstrated a successful reduction of 25% in salt intake among children and families through a school-based education programme.[20] We hypothesized that social networks could play an important role in affecting effective behaviour changes of children to reduce salt intake. Therefore, the current study determined whether the three domains of children's social networks, including peers, families and teachers, were associated with the magnitude of salt reduction among children in the intervention group.

For peer review only - http://bmjopen.bmj.com/site/about/guidelines.xhtml

METHODS

The School-EduSalt trial

School-EduSalt (**School** Based **Edu**cation Programme to Reduce **Salt**) was a cluster-randomized controlled trial carried out in 28 primary schools in an urban area of northern China (Trial registration number at ClinicalTrials.gov: NCT01821144). One class in grade 5 was selected from each school. The 28 classes were randomly allocated (1:1) to either intervention or control group. School-based salt-reduction education programme was delivered to all the children and their families in the 14 classes of the intervention group, while no specific intervention was delivered to the control group. The duration of the intervention was a school semester (≈3.5 months). Around 10 children were randomly selected from each class and two adult family members of each of these children were invited to collect two-consecutive 24-hour urine at both baseline and the end of trial. A detailed report of the study design and the main results can be found elsewhere. [20 23] Only brief methods related to this study were described here.

The children social networks study

The subjects of this study were all the schoolchildren aged 11.2 ± 0.6 (SD) years in the intervention group. The primary outcome was the score on salt-reduction behaviours (hereinafter referred to as SRB score), validated by the reduction in salt intake measured by 24h urinary sodium excretion before and after the intervention. Primary exposure measurements were factors related to the three major domains of the networks of children, including family support, peer network and teacher engagement. Children's peer networks were socio-centric networks with each of the 14 classes being a complete network, and all 603 children receiving intervention were recruited. For family and teacher network, only family support and teacher engagement were assessed due to data availability.

Participant involvement

The school principals and head teachers of the included schools were involved in the study design stage for the development of intervention activities and for the feasibility assessment

For peer review only - http://bmjopen.bmj.com/site/about/guidelines.xhtml

of the main trial, from which the present study was further developed. No participants were involved in setting the research question or the outcome measures for the present study, nor were they asked to advise on the interpretation or the writing up of this study.

Data collection

Data used in this study were collected throughout the main trial, together with an additional post-trial survey specifically designed for this study (**Figure 1**). The post-trial survey was carried out among all the children in the intervention classes nine months after the end of the intervention. In this survey, children were asked to fill out a self-administered questionnaire at school time for data on children's peer network, their recall of their family members' support for the School-EduSalt intervention program during the trial period, and behaviours related to salt reduction. Almost all children (603 out of 604) completed the survey. In the main trial, two-consecutive 24-hour urine for each child, both before and after the intervention, was collected among a random sample of 141 children to estimate the change in salt intake. [20 23] More details are given below.

Outcome assessment

The SRB score was derived from three questions on behaviours related to salt reduction that were collected in the post-trial survey. The three questions were the frequency of eating high-salt snacks (≥3 days/week, 1 to 3 days/week, almost never), the frequency of eating high-salt pickles (≥3 days/week, 1 to 3 days/week, almost never), and whether their families are taking actions to reduce salt intake (never, used to, now). Scores ranging from 1 to 3 were assigned to the answer of each question in order. The SRB score was calculated by summing up the standardized score (i.e. the z-score, to make the weight of each question comparable) from each question.

To validate SRB score, we assessed the association of SRB score with the reduction in salt intake measured by 24h urinary sodium excretion before and after the intervention among a random sample of children who made 24-hour urine collection as described above. Mixed linear models were used with school and class as random effects, with additional adjustment

For peer review only - http://bmjopen.bmj.com/site/about/guidelines.xhtml

for age, gender, and baseline body mass index. The 24h urine collections deemed incomplete were excluded from the analyses.[20] The number of children with both valid measurements of social network and completed 24-hour urine collection was 135.

Assessment of network measures

Family support for the salt-reduction intervention from each family member was assessed by asking all children in the post-trial survey (i.e. supporting, opposing, neither supporting nor opposing). Children's peer network was defined as friends within the same class. Each child (i.e. the nominator) was asked to nominate his/her friends (i.e. the nominees) from the same class who met at least two of the following conditions: (1) play together almost every day during school time; (2) often play or do homework together after school time (>3 days per week); (3) often share snacks and toys; (4) often share extracurricular books; and (5) often go to school or go home after school together. In order to take the strength of relation ties into account, we examined friendship ties weighted by the number of above conditions that were met. Peer network measures also included the size and density of the network, as well as degree centrality and peer exposure of each child. Specifically, network size is the number of children in each class. Network density is the total number of friendship connections (ties) divided by the total possible number of ties within the same class, which reflects the overall interconnectedness of the children in the same class.[24] Degree centrality is the total number of friends the child had, and it reflects the degree to which the child is connected to the network.[24] We used normalized degree centrality that was divided by the size of class in order to make it comparable across classes. Both density and degree centrality reflect the connectivity of a network, the former at network level and the latter at individual level. Peer exposure (peers' salt-reduction behaviours) of a child was calculated by summing and averaging the SRB scores of his/her friends. Teacher engagement was recorded during the intervention and was defined as full engagement (both the principle and headteacher attended all activities they had been invited to), partial engagement (attending some of the activities) and no engagement (attending none of the activities).

For peer review only - http://bmjopen.bmj.com/site/about/guidelines.xhtml

Data analysis

The association of family support with SRB score was assessed using mixed linear models, which accounted for the clustering effect of children nested within the same class, with the adjustment for age and gender.

For measures on peer networks, their association with SRB score was first assessed using mixed linear models, with the adjustment for age and gender. Peer networks of all children and their SRB scores were then visualized using network mapping techniques. Additionally, to examine whether SRB scores of the children were influenced by the SRB level of their friends (i.e. peer influence), analysis was performed from individual-focused and relation-focused facet respectively. We first built traditional (individual-focused) mixed models on children's SRB score, with children's peer exposure, age and gender as covariates. This traditional method using mixed models considered the dependence of observations, but it may not be the optimal method to address the dependence due to the overlap of peer exposures. To address this issue and make inference from network structures, we then used exponential random graph models (ERGM).[25] The ERGM of social network structure was fit for each peer network based on the difference in SRB scores between each pair of friend ties. This model explained whether the probability of the existence of friend ties between two children was associated with the similarity in SRB scores.[26] We applied the same model to each of the 14 classes and then used meta-analysis to pool the results across classes.[27 28] Fixed effect model would be used in the absence of significant heterogeneity as assessed by I^2 statistics, otherwise random-effect model would be used.

Teacher engagement was examined using methods consistent with those for family support. To additionally explore which network (family, peer and teacher) would be potentially more important in influencing the salt-reduction behaviours of children, as a secondary analysis, we examined the role of children's social networks simultaneously by including a representative factor for each network in the mixed linear model.

Sensitivity analysis

For peer review only - http://bmjopen.bmj.com/site/about/guidelines.xhtml

To test the robustness of primary findings, we performed a sensitivity analysis in a random sample of children (n=135) with completed 24h urine collection at both baseline and the end of the trial. Specifically, change in salt intake (latter-former), assessed by 24-hour urine sodium excretion, was used as the outcome. The association of three network components (i.e. peer, family and teacher network components) with change in salt intake of children were investigated using mixed models with adjustment for age, gender and baseline body mass index.

Traditional data analysis was performed in SAS version 9.4. Network properties were calculated in Ucinet.6.0 [29] while networks were visualized in NetDraw version 2.155 [24 30]. ERGM models were fit using the *statnet* package in R environment [22 25].

For peer review only

For peer review only - http://bmjopen.bmj.com/site/about/guidelines.xhtml

RESULTS

Participant Characteristics

The average age of the 603 children (boys: 50.2 %) was 11.2 (mean) ± 0.6 (SD) years old. Almost half (46%) gained full support from their families for the School-EduSalt salt-reduction program. The proportion of boys whose fathers didn't support the program was higher compared with girls (*P*=0.0035). Around 50% of the children had teachers fully engaged in the intervention program (Table 1). The average size of the classes was 43 (range: 13-77). Salt-reduction behaviours of children are described in Table 2.

Validation of the Score on Salt-Reduction Behaviours (SRB scores)

In the random sample of 135 children with complete 24h urine collection both before and after intervention, the validation analysis showed that every one-point increase in SRB score was associated with a 0.31g/d (95%CI: 0.06~0.57, *P*=0.016) greater reduction in salt intake after adjusting for age, gender and baseline body mass index. As further illustrated in Figure 2, the reduction in salt intake increased gradually from the lowest to the highest quartile of SRB score, presenting a significant linear trend (*P* trend =0.036), with a greater SRB score indicating a larger (favourable) reduction in salt intake.

Measures of social network and SRB scores

From the perspective of family networks, the SRB score of children was less with more family members not supporting salt reduction (*P* for trend<0.0001). The SRB score of children whose father didn't support salt reduction was significantly lower compared to the children whose father did (*P*<0.001). Similar association was observed for the support from mother. The magnitudes of the associations appeared smaller for the support from grandparents (Table 3).

From the perspective of peer networks, smaller size, higher density, and higher normalized degree centrality were significantly associated with a larger SRB score after adjusting for children's age and gender (Table 3). Children's peer networks were further visualized in

For peer review only - http://bmjopen.bmj.com/site/about/guidelines.xhtml

Figure 3, showing that children of greater scores (i.e. greater nodes) appeared to have denser local networks. Furthermore, traditional analysis shows that every one-unit increase in the SRB score of the peers was associated with a slight and non-significant increase of 0.07 (95%CI: -0.07 to 0.20, P=0.21) in the SRB score of children (Table 3). Consistently, according to meta-analysis of the 14 exponential random graph models built for each of the 14 networks, similarity in SRB score among friends was not found (Odds Ratio = 1.02, 95%CI: 0.93 to 1.11, *P*=0.68, Supplementary Figure S1).

In terms of teacher engagement, the SRB scores of the 311 children, from schools where both the principal and head teacher fully participated in the intervention, were significantly higher, compared to their counterparts from the other schools (difference in SRB score: 0.33; 95%CI: 0.01 to 0.64, *P*=0.043, Table 3).

When network factors from the above three domains were examined in the same model, the role of family support remained statistically significant (*P*<0.0001), The association with the degree of being connected to peers in the class, as measured by the normalized degree centrality, became borderline significant (*P*=0.13). Findings were similar for size and density of peer networks (data not shown). The association of SRB score with teacher engagement was also attenuated (Table 3).

In the final sensitivity analysis replacing SRB score with the change in salt intake measured by 24-hour urinary sodium excretion as the outcome, results were consistent with primary findings. Specifically, with more family members not supporting salt reduction, the reduction in salt intake of children tended to be less (*P* for trend=0.01). Children whose father didn't support salt reduction also had an average of 1.39 gram (95%CI: 0.38-2.41) less reduction in daily salt intake compared to the children with support from all family member (P<0.001). The role of family support remained statistically significant after further accounting for factors of peer and teacher networks. The association of change in salt intake with peer and teacher network factors were not statistically significant, with point estimates showing essentially similar patterns (Supplementary Table S1).

For peer review only - http://bmjopen.bmj.com/site/about/guidelines.xhtml

DISCUSSION

Our study is the first to explore the role of social networks in salt-reduction behaviours. We found that salt-reduction behaviours in children were related to their family support, peer networks and teacher engagements from multiple aspects. These findings offer new insights into the potential mechanisms underlying successful salt-reduction interventions in the School EduSalt trial. In this trial, interventions were implemented at the class level, with activities strategically designed to engage families, peers, teachers and school administrators. This study provides new evidence on how to design effective behaviour change programs to reduce salt intake from a social network perspective.

Our findings on the role of family networks in salt-reduction interventions were consistent with the main body of evidence that demonstrates the role of family support in shaping smoking, physical activity and dietary behaviours.[31-33] Our results also suggest that family network, especially family support, could play a more important role in shaping the behaviours of children to reduce salt intake, compared to peer and teacher networks. One explanation could be that children recruited in this study usually had meals at home and therefore their family members had more opportunities to exert a significant influence on their eating behaviours. For peer networks, both density and degree centrality, which reflect better interconnectivity of network members, were associated with favourable behaviours to reduce salt intake. It is possible that the networks that are better interconnected could provide children with more opportunities to gain peer support [34] and to influence their peers, which would in turn lead to better salt-reduction behaviours. Likewise, children with more friends within the same class may have a better sense of belonging to the class and were thus more likely to get involved in salt-reduction activities in the class. Similar inverse association between obesity and density of friend networks was also reported in an observational study.[35] By contrast, peer influence and homophily phenomena, reported for obesity [8] and smoking, [9] were not observed for salt reduction in this study. One possibility could be that children with obesity and smoking behaviours are likely to play together but children with similar salt intakes may not necessarily do so. It indicates that, unlike smoking and obesity, salt reduction

For peer review only - http://bmjopen.bmj.com/site/about/guidelines.xhtml

behaviours of a child may not influence its peer friendship, or vice versa. Furthermore, as first reported in this study, the engagement of school teachers in the intervention activities may also significantly influence the salt-reduction behaviours of children. Their role might be directly exerted through strengthening the children's attention to the intervention activities or reinforcing their beliefs in the salt-reduction messages delivered during the health education classes. It could also be mediated through their indirect influences on parents so that parents were more actively engaged in the intervention activities.

The role of social networks in shaping attitudes and behaviours related to non-communicable chronic diseases has been increasingly recognized.[36] Emerging intervention studies incorporating network components have achieved desired behaviour changes.[10-13] For example, it was reported that observing videos of heroic peers eating fruit and vegetables increased fruit consumption in children.[10] It was also reported that intervention for smoking cessation delivered by peers was more effective compared to usual care.[12] Furthermore, modifying individual's network structures may enhance the social diffusion of desired behaviours. [37] One study found that assigning intervention groups based on naturally-formed network, compared to random assignment, enhanced the effect of tobacco control among children.[11] Similarly, a nutritional intervention that targeting nominated friends of individuals was also demonstrated to increase the adoption of a nutritional intervention.[38] Nevertheless, given the difficulty in implementing effective dietary and lifestyle interventions, the evidence on incorporating individuals' social networks into behaviour change interventions is far from sufficient. This is particularly true considering that non-communicable diseases are rooted in unhealthy lifestyle behaviours. Our study, together with existing evidence,[13] suggests the great potential to promote desired behaviour changes and to effectively prevent non-communicable diseases through assessing the social network context and engaging appropriate network interventions.

Our study has several limitations. Our data on peer networks and family support were collected after the end of the trial, which limited our ability to draw causal conclusions on the association of peer network factors with salt-reduction behaviours. Recall bias is also

For peer review only - http://bmjopen.bmj.com/site/about/guidelines.xhtml

possible and it is more likely that individuals misreported less favourable support as desired support rather than the other way around, possibly resulting in underestimates of the relationships. Additionally, for family and teacher networks, we only examined family support and teacher engagement due to limited data availability. Finally, although peer exposure was not associated with individuals' behaviours to reduce salt intake in the current study, peer networks beyond the scope of the current study may play a role, such as friends beyond the class and from social media. Our study also has several strengths. This is the first study to explore the role of social network factors in salt-reduction behaviours. This study is based on a well-designed randomised controlled trial that successfully reduced salt intake in children and their families in the intervention arm by about one fourth in a school semester. Moreover, the primary outcome, i.e. SRB score, was validated by sodium intake assessed by 24h urine collection-the current gold method for measuring salt intake-in a random sample of children.[39] Though an ideal solution would be assessing behaviours using 24h urine collection for children, it is not feasible to implement this on a large scale as it is costly, resource consuming, and challenging to ensure the completeness of 24h urine collection.

CONCLUSION

Our study demonstrates that social networks may play an important role in shaping the behaviours to reduce salt intake in children. It provides new evidence for the benefit of integrating social network components into behaviour change interventions. It has a significant implication for the development of salt-reduction strategies. In a broader context, the study also sheds light on the great potential of incorporating social networks in other diet and lifestyle interventions for the prevention and control of lifestyle-related diseases.

For peer review only - http://bmjopen.bmj.com/site/about/guidelines.xhtml

Acknowledgements We thank the children and their families, the head teachers and school principals who participated in the study. We also thank all the team members from Changzhi Medical College, particularly Jianhui Yuan, Yanbo Han, Zhifang Li, Jianbin Zhang, Peifen Duan, Cailing Wei, Yanli Zhai, Ziqun Zhuo, Meng Xiao and Yunbin Zhang for their help with the data collection.

Contributors FJH, YW, XF, JM, HW and GAM designed the main study (School-EduSalt trial). YW and YM designed this sub-study and developed the analysis plan. XF, YM, JY and JZ contributed to data collection. YM, YY, TW and WX performed statistical analyses. YM, YW, FJH, WX, YY, TW, HW and GAM contributed to the interpretation of data. YM and YW took responsibility for the integrity of the data and the accuracy of the data analysis. YM wrote the first draft of the manuscript. YW, FJH, XF, JM, HW, JZ, WX, TW, YY, JY and GAM critically reviewed the manuscript. All authors approved the final version. YM and YW are guarantors.

Funding The study was funded by the UK Medical Research Council (MR/J015903/1).

Disclaimer The funder of the study had no role in the design of the study; the collection, analysis, and interpretation of the data; the writing of the manuscript; and the decision to submit the article for publication.

Participant consent Written informed consent was obtained from all the participants. For children, participant assent and parental written consent were obtained.

Competing interests All authors declare: no support from any organisation for the submitted work; no financial relationships with any organisations that might have an interest in the submitted work in the previous three years.

Ethics approval This study was approved by Peking University Health Science Centre Institutional Review Board (IRB00001052-12072) and Queen Mary University of London Research Ethics Committee (QMREC2012/81).

For peer review only

Data sharing statement The datasets for the current study are available from the corresponding author on reasonable request.

REFERENCES

1. Health effects of dietary risks in 195 countries, 1990-2017: a systematic analysis for the Global Burden of Disease Study 2017. *Lancet* 2019 doi: 10.1016/s0140-6736(19)30041-8.
2. Eckel RH, Jakicic JM, Ard JD, et al. 2013 AHA/ACC guideline on lifestyle management to reduce cardiovascular risk: a report of the American College of Cardiology/American Heart Association Task Force on Practice Guidelines. *Journal of the American College of Cardiology* 2014;63(25 Pt B):2960-84. doi: 10.1016/j.jacc.2013.11.003.
3. Wu Y, Benjamin EJ, MacMahon S. Prevention and Control of Cardiovascular Disease in the Rapidly Changing Economy of China. *Circulation* 2016;133(24):2545-60. doi: 10.1161/circulationaha.115.008728.
4. Amine E, Baba N, Belhadj M, et al. Diet, nutrition and the prevention of chronic diseases: report of a Joint WHO/FAO Expert Consultation: World Health Organization 2002.
5. GBD Risk Factors Collaborators. Global, regional, and national comparative risk assessment of 79 behavioural, environmental and occupational, and metabolic risks or clusters of risks in 188 countries, 1990-2013: a systematic analysis for the Global Burden of Disease Study 2013. *Lancet* 2015;386(10010):2287-323. doi: 10.1016/S0140-6736(15)00128-2.
6. Knapper JT, Ghasemzadeh N, Khayata M, et al. Time to Change Our Focus: Defining, Promoting, and Impacting Cardiovascular Population Health. *Journal of the American College of Cardiology* 2015;66(8):960-71. doi: 10.1016/j.jacc.2015.07.008.
7. Newson RS, Elmadfa I, Biro G, et al. Barriers for progress in salt reduction in the general population. An international study. *Appetite* 2013;71:22-31. doi: 10.1016/j.appet.2013.07.003.
8. Christakis NA, Fowler JH. The Spread of Obesity in a Large Social Network over 32 Years. *New England Journal of Medicine* 2007;357(4):370-79.
9. Christakis NA, Fowler JH. The Collective Dynamics of Smoking in a Large Social Network. *New England Journal of Medicine* 2008;358(21):2249-58.
10. Lowe CF, Horne PJ, Tapper K, et al. Effects of a peer modelling and rewards-based intervention to increase fruit and vegetable consumption in children. *European journal of clinical nutrition* 2004;58(3):510-22.
11. Valente TW, Hoffman BR, Ritt-Olson A, et al. Effects of a social-network method for group assignment strategies on peer-led tobacco prevention programs in schools. *Am J Public Health* 2003;93(11):1837-43.
12. Malchodi CS, Oncken C, Dornelas EA, et al. The effects of peer counseling on smoking cessation and reduction. *Obstet Gynecol* 2003;101(3):504-10.
13. Valente TW. Network interventions. *Science* 2012;337(6090):49-53. doi: 10.1126/science.1217330.

For peer review only - http://bmjopen.bmj.com/site/about/guidelines.xhtml

14. Campbell R, Starkey F, Holliday J, et al. An informal school-based peer-led intervention for smoking prevention in adolescence (ASSIST): a cluster randomised trial. *Lancet* 2008;371(9624):1595-602. doi: 10.1016/s0140-6736(08)60692-3.
15. Fletcher A, Bonell C, Sorhaindo A. You are what your friends eat: systematic review of social network analyses of young people's eating behaviours and bodyweight. *J Epidemiol Community Health* 2011;65(6):548-55. doi: 10.1136/jech.2010.113936.
16. Buller DB, Morrill C, Taren D, et al. Randomized trial testing the effect of peer education at increasing fruit and vegetable intake. *J Natl Cancer Inst* 1999;91(17):1491-500.
17. Marrero NM, He FJ, Whincup P, et al. Salt intake of children and adolescents in South london: consumption levels and dietary sources. *Hypertension* 2014;63(5):1026-32. doi: 10.1161/hypertensionaha.113.02264.
18. Grimes CA, Riddell LJ, Campbell KJ, et al. Dietary salt intake assessed by 24 h urinary sodium excretion in Australian schoolchildren aged 5-13 years. *Public Health Nutr* 2013;16(10):1789-95. doi: 10.1017/s1368980012003679.
19. Morinaga Y, Tsuchihashi T, Ohta Y, et al. Salt intake in 3-year-old Japanese children. *Hypertension research : official journal of the Japanese Society of Hypertension* 2011;34(7):836-9. doi: 10.1038/hr.2011.55.
20. FJ H, Y W, FengXX, et al. School based education programme to reduce salt intake in children and their families (School-EduSalt): cluster randomised controlled trial. . *BMJ* 2015;350:h770.
21. Cooper R, Soltero I, Liu K, et al. The association between urinary sodium excretion and blood pressure in children. *Circulation* 1980;62(1):97-104.
22. Hofman A, Hazebroek A, Valkenburg HA. A randomized trial of sodium intake and blood pressure in newborn infants. *JAMA* 1983;250(3):370-3.
23. He FJ, Wu Y, Ma J, et al. A school-based education programme to reduce salt intake in children and their families (School-EduSalt): protocol of a cluster randomised controlled trial. *BMJ open* 2013;3(7):e003388-e88. doi: 10.1136/bmjopen-2013-003388.
24. Hawe P, Webster C, Shiell A. A glossary of terms for navigating the field of social network analysis. *J Epidemiol Community Health* 2004;58(12):971-5.
25. Goodreau SM. Advances in Exponential Random Graph (p*) Models Applied to a Large Social Network. *Social networks* 2007;29(2):231-48. doi: 10.1016/j.socnet.2006.08.001.
26. McPherson M, Smith-Lovin L, Cook JM. Birds of a feather: Homophily in social networks. *Annual Review of Sociology* 2001:415-44.
27. Snijders TAB, Baerveldt C. A multilevel network study of the effects of delinquent behavior on friendship evolution. *The Journal of Mathematical Sociology* 2003;27(2-3):123-51. doi: 10.1080/00222500305892.
28. Valente TW, Fujimoto K, Chou C-P, et al. Adolescent Affiliations and Adiposity: A Social Network Analysis of Friendships and Obesity. *Journal of Adolescent Health* 2009;45(2):202-04.
29. Borgatti SP, Everett MG, Freeman LC. Ucinet 6 for Windows: Software for Social Network Analysis: Analytic Technologies 2002.
30. Borgatti, S.P. 2002. NetDraw: Graph Visualization Software. Harvard: Analytic Technologies.

For peer review only - http://bmjopen.bmj.com/site/about/guidelines.xhtml

31. Berkman LF, Glass T, Brissette I, et al. From social integration to health: Durkheim in the new millennium. *Soc Sci Med* 2000;51(6):843-57.
32. Scholz U, Ochsner S, Hornung R, et al. Does social support really help to eat a low-fat diet? Main effects and gender differences of received social support within the Health Action Process Approach. *Applied psychology Health and well-being* 2013;5(2):270-90. doi: 10.1111/aphw.12010.
33. Niermann CY, Kremers SP, Renner B, et al. Family Health Climate and Adolescents' Physical Activity and Healthy Eating: A Cross-Sectional Study with Mother-Father-Adolescent Triads. *PloS one* 2015;10(11):e0143599. doi: 10.1371/journal.pone.0143599.
34. Gesell SB, Barkin SL, Valente TW. Social network diagnostics: a tool for monitoring group interventions. *Implement Sci* 2013;8:116.
35. Lee WJ, Youm Y, Rhee Y, et al. Social network characteristics and body mass index in an elderly Korean population. *Journal of preventive medicine and public health = Yebang Uihakhoe chi* 2013;46(6):336-45. doi: 10.3961/jpmph.2013.46.6.336.
36. Luke DA, Harris JK. Network analysis in public health: history, methods, and applications. *Annu Rev Public Health* 2007;28:69-93.
37. Laranjo L, Arguel A, Neves AL, et al. The influence of social networking sites on health behavior change: a systematic review and meta-analysis. *Journal of the American Medical Informatics Association* 2015;22(1):243-56. doi: 10.1136/amiajnl-2014-002841.
38. Kim DA, Hwong AR, Stafford D, et al. Social network targeting to maximise population behaviour change: a cluster randomised controlled trial. *The Lancet* 2015;386(9989):145-53. doi: https://doi.org/10.1016/S0140-6736(15)60095-2.
39. McLean RM. Measuring Population Sodium Intake: A Review of Methods. *Nutrients* 2014;6(11):4651-62. doi: 10.3390/nu6114651.

For peer review only

For peer review only - http://bmjopen.bmj.com/site/about/guidelines.xhtml

Table 1. Participant characteristics

Characteristics	All (N=603)
Age (years)	11.2±0.6
Boy, n (%)	303 (50.2)
**Family support,** n (%)	
Family members not supporting salt reduction (number)	
>=3	72 (12.0)
2	91 (15.1)
1	160 (26.6)
0	279 (46.3)
Family members not supporting salt reduction (roles)*	
None	279 (46.3)
Father	154 (25.6)
Mother	79 (13.1)
Grandmother	90 (14.9)
Grandfather	74 (12.3)
**Peer network measures, n (%)**	
Network size (number of children within the class) †	43±16
Network density (in %) †	11±7
Number of friends	4 (2-5)
Normalized degree centrality (in %) ‡	24 (12-43)
Peer exposure (score on salt-reduction behaviors)	0.04 (-0.70-0.80)
**Teacher engagement, n (%)**	
Full	311 (51.6)
Partial	292 (48.4)

Data are shown in the format of mean ± SD, median (P25 - P75), and n (%).
* The overall proportion exceeded 100% as there were cases in which two or more family members did not support salt reduction, i.e. these categories were not mutually exclusive.
† Measures at cluster (class) level. Density of network is the total number of friendship connections (ties) divided by the total possible number of friendship ties within the same class.
‡ Normalized degree centrality is the number of friends within the class divided by the size of class.

For peer review only - http://bmjopen.bmj.com/site/about/guidelines.xhtml

Table 2. Description of children's salt-reduction behaviors

Questions related to salt reduction (score assigned for each answer)	All (N=603)
**Q1.Frequency of eating pickles, N(%)**	
Almost never (=3)	446 (74.0)
1~3days/week (=2)	147 (24.3)
≥3 days/week(=1)	10 (1.7)
**Q2.Frequency of eating high-salt snacks, N(%)**	
Almost never (=3)	166 (27.6)
1~3days/week (=2)	358 (59.3)
≥3 days/week(=1)	78 (13.0)
**Q3.Family's action on salt reduction, N(%)**	
Have been reducing salt (=3)	401 (66.6)
Used to (=2)	172 (28.6)
Never (=1)	29 (4.8)
**Sum of Q1-Q3 scores, N(%)**	
7-9	95 (15.8)
4-6	482 (80.2)
0-3	24 (4.0)
**SRB score (the sum of standardized scores), (Mean ± SD)**	0±1.9

For peer review only

For peer review only - http://bmjopen.bmj.com/site/about/guidelines.xhtml

Table 3 Association of SRB score with social network measures among all 603 children

Network measures	Regression coefficient (95%CI) *	*P* value
**Family support**		
Family member not supporting salt reduction (number) †		
>=3	-1.1 (-1.6 to -0.6)	<0.0001
2	-1.0 (-1.4 to -0.5)	<0.0001
1	-0.6 (-0.9 to -0.2)	0.003
0	Reference	-
Family member not supporting salt reduction (roles)		
Father	-1.3 (-1.6 to -0.9)	<0.0001
Mother	-1.2 (-1.8 to -0.6)	0.002
Grandmother	-0.7 (-1.4 to -0.1)	0.024
Grandfather	-0.8 (-1.5 to 0.02)	0.06
None	Reference	-
**Peer network measures**		
Network size (number of children within the class)	-0.01 (-0.02 to -0.002)	0.026
Network density (in %)	0.94 (0.06 to 1.82)	0.036
Normalized degree centrality (in %)	0.5 (0.01 to 0.99)	0.044
Peer exposure (score on salt-reduction behaviors)	0.07 (-0.07 to 0.20)	0.205
**Teachers' engagement**		
Partial	-0.33 (-0.64 to -0.01)	0.043
Full	Reference	-
**Joint association**		
Family support (partial versus full) ‡	-0.82 (-1.13 to -0.52)	<0.0001
Peer network (normalized degree centrality)	0.37 (-0.11 to 0.85)	0.13
Teacher engagement (partial versus full)	-0.25 (-0.56 to 0.07)	0.20

*Linear regression coefficients for corresponding network measures in the mixed models on SRB score.
†*P* for trend<0.0001.
‡ Full support was defined as all family members supporting salt reduction. Partial support was defined as at least one family member not supporting salt reduction.

For peer review only

Figure legends

Figure 1. School-EduSalt network study design.

Figure 2. Change in salt intake measured by 24h urinary sodium across the quantiles of SRB score in children.

Figure 3. Visualization of peer networks in the 14 intervention schools.

Supplementary Figure S1. The association between the existence of friend ties and similarity in SRB score.

For peer review only

For peer review only - http://bmjopen.bmj.com/site/about/guidelines.xhtml

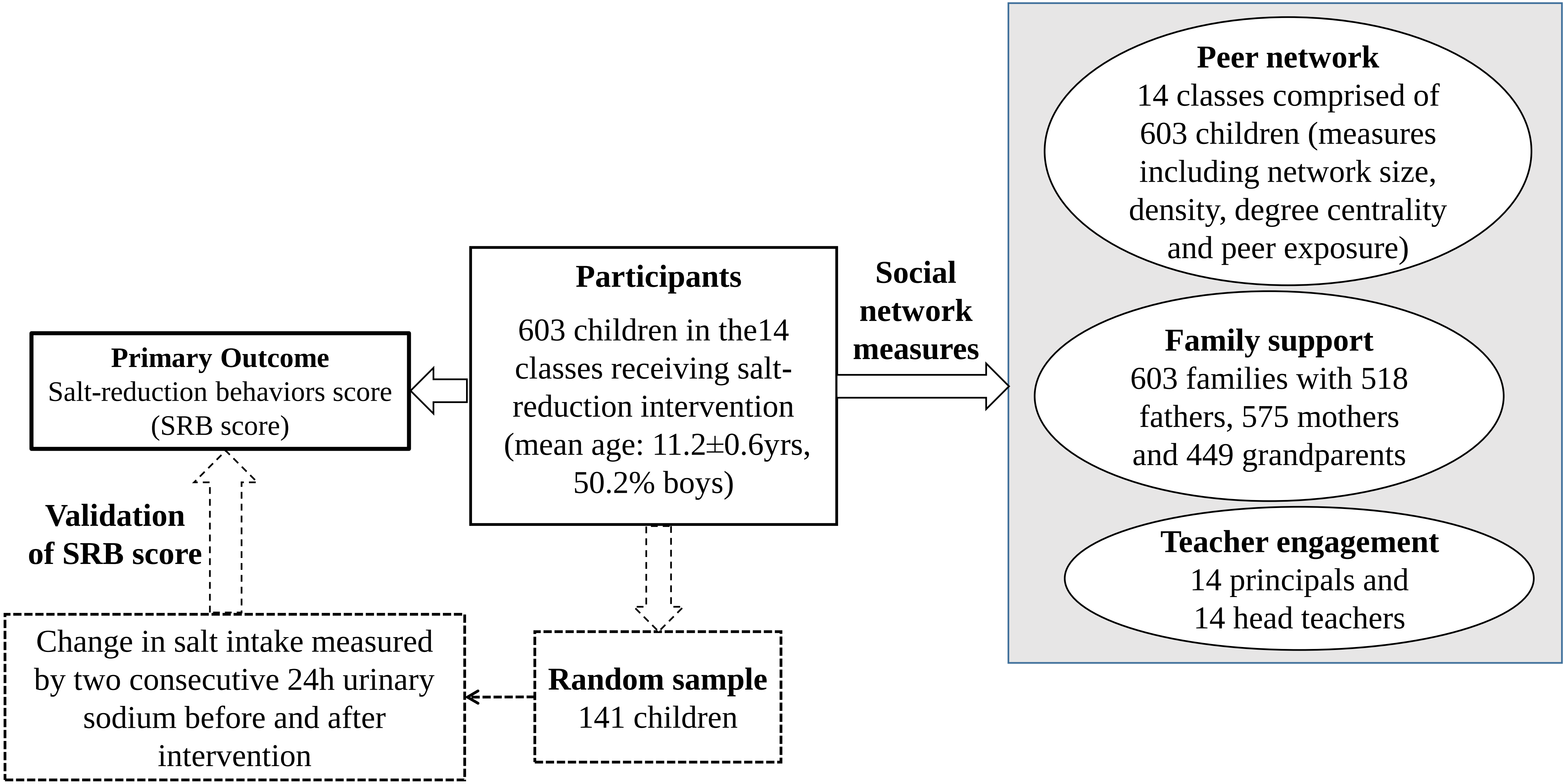

For peer review only - http://bmjopen.bmj.com/site/about/guidelines.xhtml

Figure 1 Study design

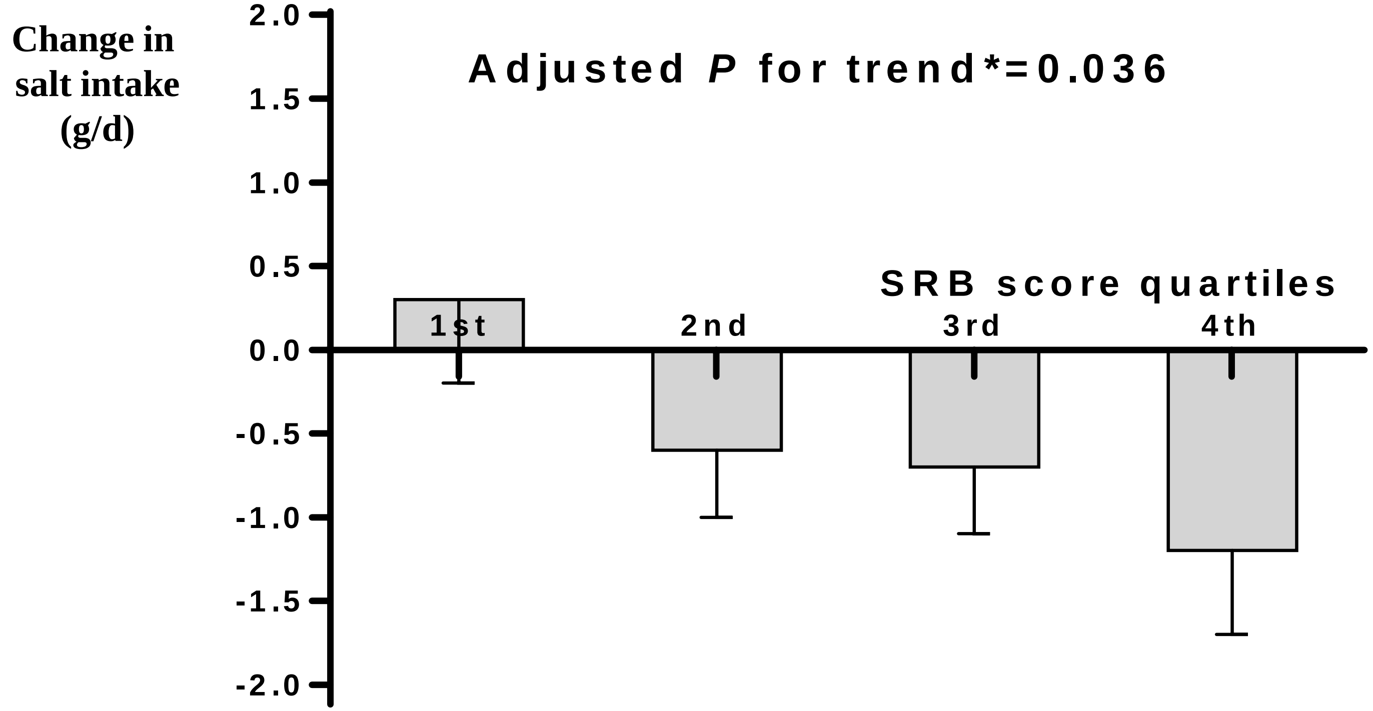

Figure 2. Change in salt intake measured by 24-hour urinary sodium excretion across the quartiles of the score on salt –reduction behaviors (SRB score).
*Adjusting for age, sex and body mass index.

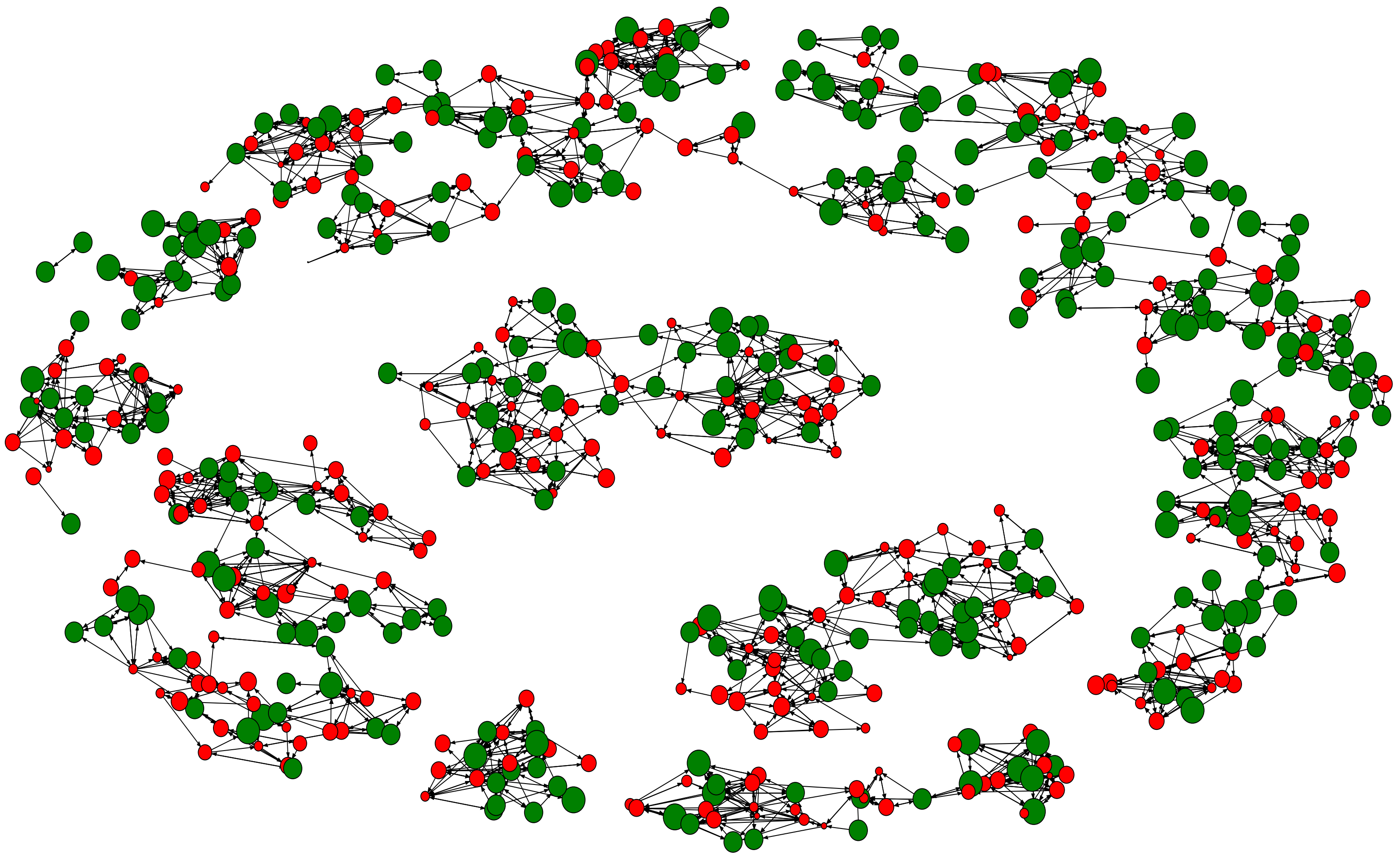

Figure 3. Visualization of peer networks in the 14 intervention schools

*Each node represents a child. The tie between two nodes denotes a friend relation. The size of each node is proportional to the score on salt-reduction behaviour (A higher score indicates a greater reduction in salt intake). Nodes with score above the median were painted in green colour and those below the median were in red.

Online Supplementary Material

Figure S1. The association between the existence of friend ties and similarity in SRB score

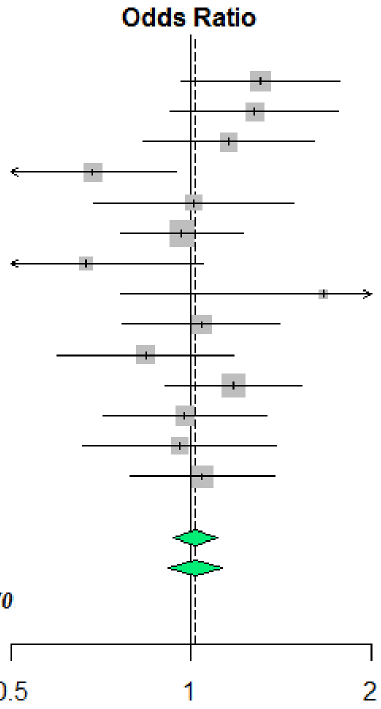

Networks	Estimate	Standard error	OR	95%-CI	W(fixed)	W(random)
1	0.27	0.1561	1.31	[0.96; 1.78]	7.9%	8.0%
2	0.24	0.1662	1.28	[0.92; 1.77]	7.0%	7.3%
3	0.15	0.1680	1.16	[0.83; 1.61]	6.8%	7.2%
4	-0.38	0.1627	0.69	[0.50; 0.94]	7.3%	7.5%
5	0.01	0.1965	1.01	[0.69; 1.49]	5.0%	5.7%
6	-0.04	0.1196	0.96	[0.76; 1.22]	13.5%	11.0%
7	-0.40	0.2320	0.67	[0.42; 1.05]	3.6%	4.4%
8	0.51	0.4001	1.67	[0.76; 3.65]	1.2%	1.7%
9	0.04	0.1537	1.04	[0.77; 1.41]	8.2%	8.1%
10	-0.17	0.1739	0.84	[0.60; 1.18]	6.4%	6.8%
11	0.16	0.1330	1.18	[0.91; 1.53]	10.9%	9.8%
12	-0.02	0.1608	0.98	[0.71; 1.34]	7.5%	7.6%
13	-0.04	0.1905	0.96	[0.66; 1.39]	5.3%	6.0%
14	0.04	0.1422	1.04	[0.79; 1.38]	9.5%	9.0%
**Overall effect**			**1.02**	**[0.93; 1.11]**	**100%**	**--**
**Random effects model**			**1.02**	**[0.91; 1.13]**	**--**	**100%**

Heterogeneity: I-squared=30%, tau-squared=0.0117, p=0.1370

Test for overall effect (fixed effect): p=0.6842

*Odds ratio of the existence of a friend tie associated with one-unit increase in the similarity of SRB score between each pair of children.

Supplementary Table S1. Association of social network measures with reduction in salt intake measured by 24hUNa in a random sample of 135 children [*]

Network measures	reduction in the amount of salt intake (95%CI), g/d †	*P* value
**Family support**		
Family member not supporting salt reduction (number) ‡		
>=3 (n=9)	-1.9 (-3.4 to -0.3)	0.018
2 (n=14)	-0.5 (-1.8 to 0.8)	0.466
1 (n=28)	-1.0 (-2.0 to -0.01)	0.049
0 (n=84)	Reference	-
Family member not supporting salt reduction (roles)		
Father (n=24)	-1.4 (-2.4 to -0.4)	0.004
Mother (n=9)	0.1 (-1.9 to 2.2)	0.89
Grandmother (n=16)	-1.3 (-2.9 to 0.4)	0.13
Grandparents (n=11)	-0.7 (-2.4 to 1.1)	0.42
None (n=84)	Reference	-
**Peer network measures**		
Network size	-0.01 (-0.05 to 0.03)	0.657
Network density	0.9 (-1.30 to 3.1)	0.410
Normalized degree centrality	-0.1 (-1.2 to 0.9)	0.825
**Teachers' engagement**		
Partial (n=56)	-0.6 (-1.4 to 0.2)	0.142
Full (n=79)	Reference	-
**Joint association**		
Family support (partial *versus* full)	-0.8 (-1.5 to -0.02)	0.046
Peer network (normalized degree centrality)	-0.5 (-1.3 to 0.4)	0.283
Teacher engagement (partial *versus* full)	-0.5 (-1.3 to 0.2)	0.172

*. 24hUNa=24-hour urine sodium excretion.

†. Reduction in salt intake indicates the change in salt intake from baseline to the end of trial (post-pre). The negative values reflect a smaller reduction in salt intake, after adjusting for age, sex, baseline body mass index and clustering effect of children nested within the same class in mixed linear models.

‡. *P* for trend=0.01. As an example, the interpretation of -1.87 (-3.41 to -0.32) for children who had ≥3 family members not supporting salt reduction, their reduction in salt intake (g/d) was 1.87g less per day, compared to their counterparts whose family supported salt reduction.

For peer review only

STROBE Statement—Checklist of items that should be included in reports of ***cross-sectional studies***

	Item No	Recommendation
**Title and abstract (√, page1) (√, page 2)**	1	(*a*) Indicate the study's design with a commonly used term in the title or the abstract
		(*b*) Provide in the abstract an informative and balanced summary of what was done and what was found
**Introduction**		
Background/rationale **(√, page 4)**	2	Explain the scientific background and rationale for the investigation being reported
Objectives **(√, page 4)**	3	State specific objectives, including any prespecified hypotheses
**Methods**		
Study design **(√, page 5)**	4	Present key elements of study design early in the paper
Setting **(√, page 5-6)**	5	Describe the setting, locations, and relevant dates, including periods of recruitment, exposure, follow-up, and data collection
Participants **(√, page 5 and figure 1)**	6	(*a*) Give the eligibility criteria, and the sources and methods of selection of participants
Variables **(√, page 6-7)**	7	Clearly define all outcomes, exposures, predictors, potential confounders, and effect modifiers. Give diagnostic criteria, if applicable
Data sources/ measurement **(√, page 5-7)**	8*	For each variable of interest, give sources of data and details of methods of assessment (measurement). Describe comparability of assessment methods if there is more than one group
Bias **(√, page 8)**	5	Describe any efforts to address potential sources of bias
Study size **(NA)**	10	Explain how the study size was arrived at
Quantitative variables **(√, page 7-9)**	11	Explain how quantitative variables were handled in the analyses. If applicable, describe which groupings were chosen and why
Statistical methods **(√, page 7-9)**	12	(*a*) Describe all statistical methods, including those used to control for confounding
**(Page 10, subgroup analysis)**		(*b*) Describe any methods used to examine subgroups and interactions
		(*c*) Explain how missing data were addressed
**(NA)**		(*d*) If applicable, describe analytical methods taking account of sampling strategy
**(√, Page 9)**		(*e*) Describe any sensitivity analyses
**Results**		
Participants **(√, page 5, page 10 and figure 1)**	13*	(a) Report numbers of individuals at each stage of study—eg numbers potentially eligible, examined for eligibility, confirmed eligible, included in the study, completing follow-up, and analysed
		(b) Give reasons for non-participation at each stage
		(c) Consider use of a flow diagram
Descriptive data **(√, page 8， figure 1 and table 1)**	14*	(a) Give characteristics of study participants (eg demographic, clinical, social) and information on exposures and potential confounders
**(Figure 1)**		(b) Indicate number of participants with missing data for each variable of interest

For peer review only - http://bmjopen.bmj.com/site/about/guidelines.xhtml

Outcome data **(NA)**	15*	Report numbers of outcome events or summary measures
Main results **(√)** **(√, table 1~3)**	16	(*a*) Give unadjusted estimates and, if applicable, confounder-adjusted estimates and their precision (eg, 95% confidence interval). Make clear which confounders were adjusted for and why they were included
		(*b*) Report category boundaries when continuous variables were categorized
**(NA)**		(*c*) If relevant, consider translating estimates of relative risk into absolute risk for a meaningful time period
Other analyses **(Page11, table S1)**	17	Report other analyses done—eg analyses of subgroups and interactions, and sensitivity analyses
**Discussion**		
Key results **(√, page 12)**	18	Summarise key results with reference to study objectives
Limitations **(√, page 14)**	19	Discuss limitations of the study, taking into account sources of potential bias or imprecision. Discuss both direction and magnitude of any potential bias
Interpretation **(√, page 11-14)**	20	Give a cautious overall interpretation of results considering objectives, limitations, multiplicity of analyses, results from similar studies, and other relevant evidence
Generalisability **(√, page 14)**	21	Discuss the generalisability (external validity) of the study results
**Other information**		
Funding **(√, page 15)**	22	Give the source of funding and the role of the funders for the present study and, if applicable, for the original study on which the present article is based

*Give information separately for exposed and unexposed groups.

Note: An Explanation and Elaboration article discusses each checklist item and gives methodological background and published examples of transparent reporting. The STROBE checklist is best used in conjunction with this article (freely available on the Web sites of PLoS Medicine at http://www.plosmedicine.org/, Annals of Internal Medicine at http://www.annals.org/, and Epidemiology at http://www.epidem.com/). Information on the STROBE Initiative is available at www.strobe-statement.org.

For peer review only
